# Comparative mitogenomic analysis of *Sporisorium reilianum* f. sp. *zeae* suggests recombination events during its evolutionary history

**DOI:** 10.3389/fphys.2024.1264359

**Published:** 2024-09-06

**Authors:** Hector Mendoza, Emma A. Lamb, Joshua Thomas, Derica Goncalves Tavares, Luke A. Schroeder, Christian Müller, Nisha Agrawal, Jan Schirawski, Michael H. Perlin

**Affiliations:** ^1^ Department of Biology, Program on Disease Evolution, University of Louisville, Louisville, KY, United States; ^2^ Matthias Schleiden Institute - Genetics, Friedrich-Schiller University Jena, Jena, Germany

**Keywords:** smut fungi, mitochondria, mitogenome evolution, population mitogenomics, horizontal gene transfer

## Abstract

**Introduction:**

Modern understanding of the concept of genetic diversity must include the study of both nuclear and organellar DNA, which differ greatly in terms of their structure, organization, gene content and distribution. This study comprises an analysis of the genetic diversity of the smut fungus *Sporisorium reilianum* f. sp. *zeae* from a mitochondrial perspective.

**Methods:**

Whole-genome sequencing data was generated from biological samples of *S. reilianum* collected from different geographical regions. Multiple sequence alignment and gene synteny analysis were performed to further characterize genetic diversity in the context of mitogenomic polymorphisms.

**Results:**

Mitochondria of strains collected in China contained unique sequences. The largest unique sequence stretch encompassed a portion of *cox1*, a mitochondrial gene encoding one of the subunits that make up complex IV of the mitochondrial electron transport chain. This unique sequence had high percent identity to the mitogenome of the related species *Sporisorium scitamineum* and *Ustilago bromivora*.

**Discussion:**

The results of this study hint at potential horizontal gene transfer or mitochondrial genome recombination events during the evolutionary history of basidiomycetes. Additionally, the distinct polymorphic region detected in the Chinese mitogenome provides the ideal foundation to develop a diagnostic method to discern between mitotypes and enhance knowledge on the genetic diversity of this organism.

## 1 Introduction

Many studies on genetic diversity focus on nuclear DNA (nDNA) and often ignore organellar DNA (oDNA) which, in contrast to nDNA, differs significantly in structure, organization, size, gene distribution and content, and mutation rate (Burger et al., 2003). Given the importance of organelles that still preserve their own DNA, like chloroplasts and mitochondria, the concept of genetic diversity studies should include close examination of such oDNAs to complement the earlier unidimensional approach. Moreover, recent mitogenomic studies have emphasized the importance of mitonuclear (bigenomic) communication in the regulation of gene expression (Rodley et al., 2012; Fetterman and Ballinger, 2019), highlighting the need for both nuclear and mitochondrial perspectives for a broader and unhindered understanding of an organism’s genetic structure and evolutionary history.

Mitochondrial genome evolution diverges significantly among eukaryotes in terms of size, intron abundance, gene content and order, and rate of mutation (Bullerwell and Gray, 2004). Fungal mitogenomes are of particular interest, as they have remained largely unexplored and have the potential to uncover additional mysteries of organelle evolution. A study in 2012 developed a classification system based on mitochondrial DNA (mtDNA), emphasizing intron abundance, size, shape and organization (Kolesnikov and Gerasimov, 2012). Accordingly, fungal mtDNAs display diversity types 2, 3 and 5, with gene content largely conserved, but with high order variability. Interestingly, fungal mtDNA contains similarities to plant mtDNA, displaying more signals of recombination and highly variable intron content which, in turn, influences mitogenome sizes (Aguileta et al., 2014; Stukenbrock and Dutheil, 2018; Fonseca et al., 2021; Formaggioni et al., 2021). Fungal introns may also exhibit autonomous proliferation via self-splicing and sequence homing, facilitated by homing endonucleases (HEs) that catalyze site-specific intron integration (Megarioti and Kouvelis, 2020). Finally, fungal mtDNA is also characterized by its highly variable distribution and editing of mitochondrial tRNAs, which allows fungi to participate in extremely rare horizontal gene transfer events (Cedergren et al., 1985). These special features of fungal mtDNA provide additional molecular markers that can be used in the study of genetic diversity and evolutionary history.

The current study examines mitogenomic diversity in the context of the inheritance mechanisms that determine its distribution in sexually reproducing eukaryotes. *Sporisorium reilianum* f. sp. *zeae* (SRZ), the causative agent of head smut disease in maize, is an excellent biological system from which to study mitochondrial inheritance patterns, given its tetrapolar mating type system (Schirawski et al., 2005) and proposed degradation-mediated uniparental inheritance mechanism, analogous to that studied in the related species, Ustilago maydis (Fedler et al., 2009; Mahlert et al., 2009). A relatively recent study confirmed that the inheritance system observed in *U. maydis* follows a uniparental pattern through the collection and analysis of mitogenomic data from samples distributed throughout Mexico, of which 75% displayed the mitotype of the published a2 strain (Holliday, 1961; Jimenez-Becerril et al., 2018). The study also hints at factors influencing dispersion and distribution of genetically diverse populations across the region.

Here, we introduce a mitogenomic approach based on whole genome sequencing (WGS) data and gene synteny analysis, for the identification of polymorphic regions in the mitogenomes of SRZ strains. The fungal samples chosen for the study were of Chinese or German origin, as it is highly improbable that their natural populations would have encountered each other, hence leaving the respective mitogenomic diversities intact. The bioinformatic analysis revealed at least 16 positions of point mutations throughout the mtDNA. Most notably, a massive deletion in the *cox1* gene of Chinese SRZ mtDNA was initially detected and predicted to have serious consequences on its corresponding polypeptide and, in turn, on mitochondrial maintenance and function. Further investigation clarified that what had appeared to be a massive deletion was, in fact, a unique insertion of DNA sequence not found in the SRZ genomic database. This region, unique to the Chinese isolates, provided an optimal region from which to design a simple and effective PCR-based diagnostic methodology to discern between German and Chinese SRZ mitogenomes. The bioinformatic data generated in this study introduces a new evolutionary context from which to study mitogenomic diversity in SRZ, in addition to providing the foundation for the verification of the predicted uniparental pattern of mitochondrial inheritance in SRZ.

## 2 Materials and methods

### 2.1 Strains and growth conditions

The SRZ strains used in this study are listed in Table 1 and were collected in regions of Germany and China. The strains were selected due to their genotypes that allow for compatible crossing (different alleles for both *a* and *b* mating type loci), as well as their different geographic origins to further increase the probability of mitochondrial polymorphisms. Haploid strains were grown in potato dextrose (PD) broth on a rotary shaker at 200 rpm at 28°C or on solid PD agar at 28°C. Strains were maintained in PD glycerol (20%) medium at −80°C for long-term storage or on PD agar at 4°C for no longer than 7 days.

**TABLE 1:** Strains used in this study.

Strain	Genotype	Geographic Origin^a^
SRZ1	a1b1	Hohenheim, Germany
SRZ2	a2b2	Hohenheim, Germany
SRZCXI2	a3b3	China “sample 1”
SRZCXII2	a3b1	China “sample 2”
SRZCXI3	a2b3	China “sample 3”
SRZ55III10	a1b2	From SRZ1 x SRZ2 teliospores

^a^ Schirawski et al., 2005.

### 2.2 Molecular techniques and bioinformatic analysis

DNA isolation from SRZ haploid cultures was carried out as previously described for *U. maydis* (Schulz et al., 1990). DNA isolation from teliospores employed the MagBeads FastDNA^®^ Kit (MP Biomedical, Irvine, California) and used 100–500 mg of the spores per extraction. WGS was performed by CD Genomics (Shirley, NY, United States) using the Illumina HiSeq Sequencing Platform. The raw sequencing data were then assembled and aligned in reference to the annotated mtDNA sequence of SRZ2 (a2b2) (NCBI Accession No. GCA_000230245.1; NCBI Accession No. FQ311469.1) in SnapGene 5.3.2 (Insightful Science, LLC).

Gene synteny analysis was performed based on the concatenated alignment of fourteen mitochondrial protein-coding genes: *atp6, atp8, atp9*, *cob*, *cox1*, *cox2*, *cox3, nad1, nad2, nad3, nad4, nad4L, nad5* and *nad6*. Confirmation of putative polymorphic regions was achieved with sequential Sanger sequencing (“primer walking”) (Eurofins, Louisville, KY, United States). Protein sequence alignments were performed by Multiple Sequence Comparison by Log-Expectation (MUSCLE) in SnapGene 5.3.2 (Insightful Science, LLC).

Polymerase chain reaction (PCR) was carried out in a T100 Thermal Cycler (Bio-Rad Laboratories) with Ex Taq Hot Start DNA Polymerase (TaKaRa Bio United States, Inc), PrimeSTAR Max DNA Polymerase (TaKaRa Bio United States, Inc) or DreamTaq Hot Start PCR Master Mix (Thermo Fisher Scientific). PCR cycling conditions for the Hot Start polymerases involved an initial denaturation step at 94°C for 4 min, followed by 35 cycles of a three-step process (denaturation at 94°C for 30 s, annealing at 58°C–62°C for 30 s and extension at 72°C for 1 min per 1 kb of anticipated product) and a final extension step at 72°C for 10 min. PCR cycling conditions for PrimeSTAR Max DNA Polymerase involved an initial denaturation step at 98°C for 2 min, followed by 35 cycles of a three-step process (denaturation at 98°C for 10 s, annealing at primer 58°C–62°C for 15 s and extension at 72°C for 30–45 s) and a final extension at 72°C for 5 min. Primers used in this study are listed in Supplementary Table S1 and the relative locations of primers used in analysis of the cox1 gene are shown in Supplementary Figure S1). Purification of DNA from agarose gels was achieved using the Zymoclean Gel DNA Recovery Kit (Zymo Research, Irvine, CA) and followed the manufacturer’s instructions. RNA isolation from SRZ strains grown on potato dextrose agar (PDA) at 28°C for 48 h used the Monarch Total RNA Miniprep Kit (New England Biolabs, Ipswich, MA). cDNA was synthesized with SuperScript™ IV First-Strand Synthesis System (Invitrogen/Thermo Fisher, Waltham, MA).

### 2.3 Mating and maize infections

Strains were grown in 50 mL of PD broth on a rotary shaker at 28°C until an OD600 of 0.5–0.8 was reached. Cells were harvested by centrifugation at 3,500 rpm and resuspended in ddH_2_O to a final OD600 of 2. The desired strain combinations were then mixed in a 1:1 ratio and used to inoculate 7-day old Tom Thumb maize (High Morning Organic Seeds) seedlings. Disease evaluation was performed 7–8°weeks post infection (wpi), in which plant height was recorded and symptoms were scored for the calculation of disease severity indexes (Ghareeb et al., 2019). One-way ANOVA followed by Tukey’s Multiple Comparison test was performed in GraphPad Prism 9.2.0 (GraphPad Software, LLC) to determine statistical significance (*p* < 0.05). Once the spores were fully formed on the infected Tom Thumb plants, they were collected and placed in envelopes. The envelopes were incubated for 48 h at 28°C until completely dry. The spores were then shaken out of the infected cobs or tassels and placed in sterile containers for further use. Teliospores from infections were used for DNA isolation and were also germinated on YPS agar. In order to assure sufficient separation of resulting colonies, 100 μL of teliospores was added to 900 μL of sterile dH_2_O; this suspension was then diluted 1:100 in sterile dH_2_O. A 100 μL aliquot of this final 1:100 dilution was spread onto YPS plates, which were incubated for 48–72 h at 28°C until individual colonies were observed. At least 10 colonies from each set of germinated teliospores were picked onto fresh YPS agar, and DNA was isolated from a minimum of 4 purified colonies from each germinated teliospore source.

## 3 Results

### 3.1 Gene synteny analysis reveals genetic variations among SRZ strains

**FIGURE 1:**
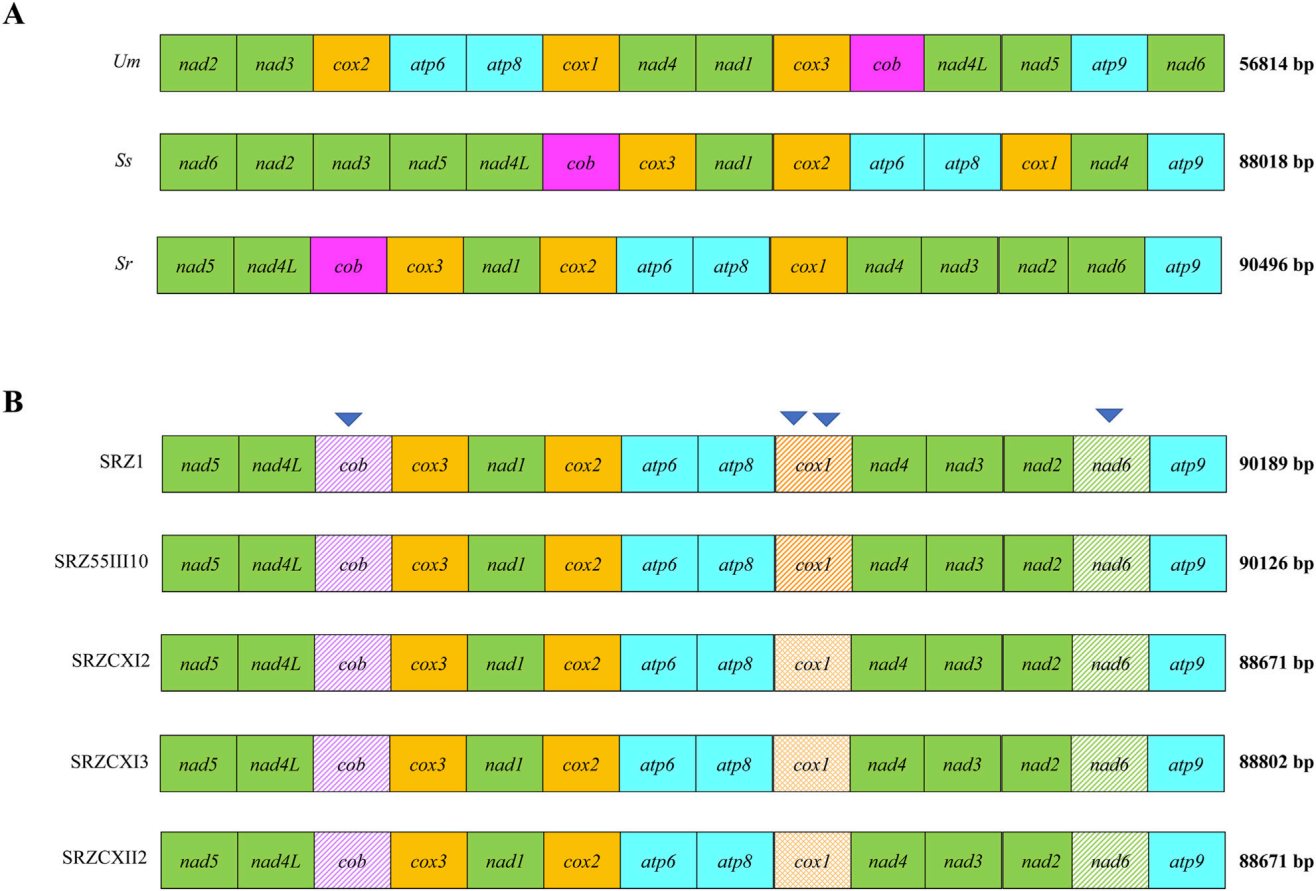
Comparison of concatenated alignments of 14 mitochondrial protein-coding genes of different fungi **(A)** or strains **(B)** were made based on **(A)** annotated reference mitogenomes (*Um* = *Ustilago maydis* (NCBI Accession No. NC_008368.1), *Ss* = *S. scitamineum* (NCBI Accession No. CP010939), *Sr* = *S. reilianum* (NCBI Accession No. FQ311469.1)) and **(B)** the raw WGS data obtained in this study. Mitogenome sizes (bp) are indicated next to each alignment. The presence of unique polymorphisms in a single gene is indicated by shade variation. Inverted triangles are used to indicate polymorphisms within the coding regions of *cob*, *nad6*, and *cox1* **(B)**.

To assess the diversity of mitochondrial genomes of *S. reilianum*, we selected strains isolated from teliospores collected on maize in Germany and China (Schirawski et al., 2005; Table 1), as the respective natural populations are expected not to have recently encountered each other, hence leaving the respective mitogenomic diversities intact. Selected strains were subjected to whole genome sequencing, and the mitochondrial genome sequences were assembled. First, sequence polymorphisms throughout the mitogenomes were identified (Table 2). Next, to compare natural diversity of mitochondrial genomes of very different sizes, we decided to focus on protein-coding genes, including their introns. Thus, we generated concatenated sequences of fourteen mitochondrially-encoded genes and used these for alignment. Alignment of the mitochondrial genes of SRZ2, *U. maydis* and *S. scitamineum* revealed diversity in gene order between the different organisms (Figure 1A), while mitochondrial gene order of the different strains of *S. reilianum* was conserved (Figure 1B). Mitochondrial genome sizes of strains of German origin (SRZ1 and SRZ55III10) were very close to that of the reference strain that was also isolated from material collected in Germany (Schirawski et al., 2010). Interestingly, mitochondrial genome sizes of the strains isolated from teliospores collected in China (SRZCXI2, SRZCXI3 and SRZCXII2) were closer in size to that of *S. scitamineum* than that of SRZ (Figure 1B).

**TABLE 2:** Summary of polymorphisms detected in sequenced mitogenomes (SRZ2 used as reference).

Region Covered	SRZ1	SRZIII10	SRZCXI2	SRZCXII2	SRZCXI3
Intergenic	m.4966_4968insG	m.4966_4968insG	m.4966_4968insG	m.4966_4968insG	m.4966_4968insG
Intronic region of *cob*	m.22393_22395insT	m.22393_22395insT	m.22393_22395insT	m.22393_22395insT	m.22393_22395insT
Intergenic	ND	m.39037del	m.39037del	m.39037del	m.39037del
Intergenic	m.(42,869_42975)ins	ND	ND	ND	ND
Intergenic	m.43211_43213insG	m.43103_43105insG	m.43103_43105insG	m.43103_43105insG	m.43103_43105insG
Intronic region of *cox1*	m.46772_46774insA	m.46664_46666insA	m.46664_46666insA	m.46664_46666insA	m.46664_46666insA
Intronic region of *cox1*	m.48456del	m.48456del	m.48456del	m.48456del	m.48456del
Intronic region of *cox1*	ND	ND	ND	ND	m.(48,944_49074)ins
Spans exons 6 and 7 of *cox1*	ND	ND	m.49207_50817del	m.49207_50817del	m.49207_50817del
Intronic Region of *cox1*	m.50647del	m.50647del	ND	ND	ND
Intergenic	m68067_68069insA	m.67959_67961insA	m.66480_66482insA	m.66480_66482insA	m.66611_66613insA
Intergenic	m.68492del	m.68492del	m.68492del	m.68492del	m.68492del
Intergenic	m.68390_68391delinsTT	m.68282_68283delinsTT	m.66803_66804delinsTT	m.66803_66804delinsTT	m.66934_66935delinsTT
Intergenic	m.84589del	ND	m.84589_84590del	m.84589_84590del	m.84589_84590del
In *nad6* CDS	m.87332del	m.87332del	m.87332del	m.87332del	m.87332del
Intergenic	m.88146_88148insG	m.88013_88015insG	m.86558_86560insG	m.86558_86560insG	m.86689_86691insG

^a^ Strains (first row) are described in Table 1. Data describe the position and the type of mutation detected. The mutations were associated with the same running number (first column) when they were detected within the same region of mtDNA, although the numerical positions may vary otherwise due to the size disparities among mitogenomes. ND, not detected, ins = insertion, del = deletion, delins = substitution. Nomenclature for description of sequence variations per den Dunnen and Atonarakis (2001) and numbering refers to the corresponding position(s) of the respective mitogenome.

Data describe the position and the type of mutation detected. The mutations were associated with the same running number (second column) when they were detected within the same region of mtDNA, although the numerical positions may vary otherwise due to the size disparities among mitogenomes. ND = not detected, ins = insertion, del = deletion, delins = substitution. Nomenclature for description of sequence variations per den Dunnen and Atonarakis (2001) and numbering refers to the corresponding position(s) of the respective mitogenome.

We then analyzed nucleotide differences between the sequences of the 14 mitochondrial genes of the sequenced strains, which revealed an array of insertions, deletions, and substitutions that were grouped into 16 sites of mutation (Table 2). Notably, nucleotide differences were found in *cob*, *cox1* and *nad6* (Figure 1B) and consisted of a single base-pair insertion in *cob* (m.4315_4317T), a single base-pair deletion in *nad6* (m.642delG) and two point mutations in *cox1* (m.708_710insA, m.2237delC). The mutations in *cob* and *cox1*, as was the case with most mutations detected, occurred in non-coding regions of the mitochondrial gene sequences and were excluded from further investigation as they should have little to no effect on mitochondrial function. In contrast, the mutation in *nad6*, a gene with no introns, is predicted to cause a frameshift. Additionally, the Chinese strains (SRZCXI2, SRZCXII2 and SRZCXI3) initially appeared to harbor a massive deletion of 1,611 bp (m.2988_4598del) based on the alignments. This last sequence variation spans intronic and protein-coding regions and, if confirmed, would be expected to cause significant modifications in its respective polypeptide sequence.

### 3.2 The *nad6* mutation may comprise a frameshift mutation on the predicted polypeptide

**FIGURE 2:**
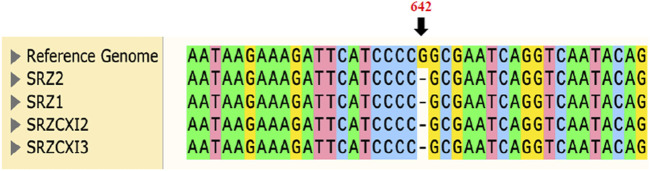
Sequence alignment of samples tested against the annotated reference genome of SRZ2 (NCBI Accession No. FQ311469.1) confirmed the point mutation previously detected for *nad6* in the raw WGS data. Only 40 bp of DNA was included for sequence alignment and position 642 of the reference genome (indicated in orange) highlights the single base-pair deletion. For representative electropherograms of these comparisons, see Supplementary Figure S2.

The *nad6* gene corresponds to a single exon of 681 bp, predicted to encode a 24.4 kDa polypeptide of 226 amino acids. Bioinformatic analysis revealed a single base-pair (m.642delG) deletion in *nad6*, at position 642 of the corresponding SRZ2 reference *nad6* DNA sequence, which would lead to a frameshift and would result in a polypeptide of only 220 amino acids. Verification of this mutation utilized amplification and sequencing of the *nad6* gene in its entirety. Sequencing was performed using opposing primers oHM121 and oHM122, in separate reactions. All samples tested indeed had the single base-pair deletion previously detected by WGS. Surprisingly, this included SRZ2, which was sequenced as control and should have been identical to the reference genome used for sequence alignment analysis (Figure 2; Supplementary Figure S1).

**FIGURE 3:**
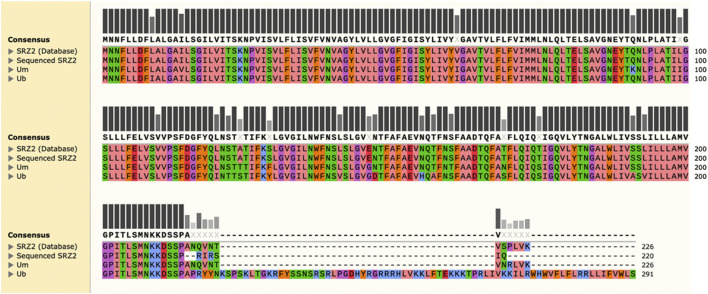
MUSCLE analysis of sequenced *nad6* against the annotated version reveals a truncation at the C-terminus of the protein. The predicted polypeptide sequences for *nad6* in *Ustilago maydis* (Um) and *Ustilago bromivora* (Ub) were included for comparison. Amino acids with similar physico-chemical properties are represented by the same color. Bars represent amino acid conservation across all sequences analyzed, with darker and taller bars corresponding to higher conservation indexes. Amino acids in bold make up the consensus sequence and represent 100% conservation in that position.

Further investigation of this mutation involved BLASTp analysis of *nad6* obtained from the sequencing results against the annotated *nad6* amino acid sequence (NCBI Accession No. CBQ72567.1) to identify the corresponding alterations. The amino acid sequence of *nad6* in the related species *U. maydis* (NCBI Accession No. YP_762704.1) and *U. bromivora* (NCBI Accession No. SAM86553.1) were included for comparison. MUSCLE analysis revealed that the mutation truncates *nad6* at the C-terminus, producing a protein of 220 amino acids, as opposed to the 226 amino acid long polypeptide predicted by the annotated amino acid sequence for the reference mitogenome (Figure 3). Interestingly, the sequence of *U. maydis nad6* is highly similar to the annotated SRZ2 *nad6*, producing a polypeptide of the same length. When *U. bromivora nad6* was included, which corresponds to a larger polypeptide (291 amino acids), it is evident that the *S. reilianum* mutation in question significantly modifies the *nad6* polypeptide, as it is missing several amino acids that are shared across the species included in the analysis.

### 3.3 Chinese mitogenomes possess an alternate version of *cox1*

**FIGURE 4:**
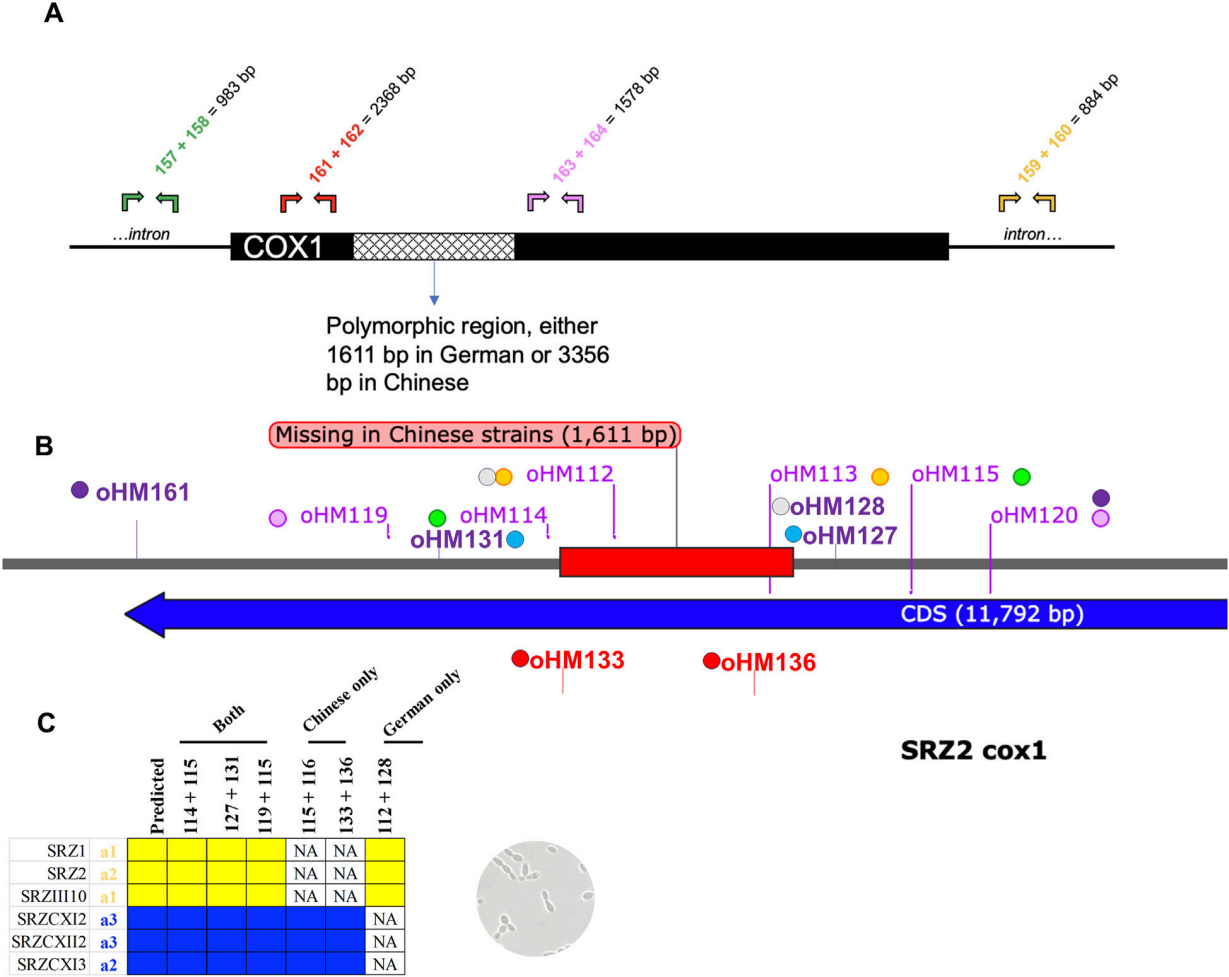
Map of diagnostic PCR for *cox1*. Sequence is based on SRZ2 reference genome. **(A)** Additional PCR experiments were carried out around the *cox1* polymorphic region. Primer pairs binding outside of the indicated region were designed to inspect mtDNA integrity. These primer combinations should work in any SRZ strain, regardless of the mitotype. Predicted amplicon sizes are indicated. **(B)** Complementary primer combinations are color coded and should yield the following amplicons: oHM114/115 = 2,507 bp in German or 1,158 bp in Chinese, oHM119/120 = 4,143 bp in German or 2,795 bp in Chinese, oHM112/128 = 1,347 bp in German only. For gel electrophoresis results, refer to Supplementary Figure S3. **(C)** Table of diagnostic primer combinations for haploid sporidial German and Chinese strains. NA, not applicable, i.e., no band was observed, as expected.

Additional PCR experiments were carried out around the *cox1* polymorphic region. Primer pairs binding outside of the indicated region were designed to inspect mtDNA integrity. These primer combinations should work in any SRZ strain, regardless of the mitotype (Figure 4A). The 1,611 bp deletion detected in Chinese strains by WGS was used to perform diagnostic PCR with three primer combinations (Figure 4B), which bind either outside or within the region in which the deletion was originally detected. For the primer combination oHM114/115, the resulting amplicon should be 2,507 bp or 1,158 bp in size for German and Chinese strains, respectively, and should be discernable after agarose gel electrophoresis. Surprisingly, all Chinese strains produced a band that was significantly larger in size (Supplementary Figure S3A). Additional primer pairs oHM119/120 (expected amplicon: 4,143 bp in German strains and 2,795 bp in Chinese strains; Supplementary Figure S3B) were used to confirm this deviation of the Chinese strains from their expected amplicon sizes.

The unexpected bands produced in the Chinese strains with the diagnostic primer pairs, as well as the expected bands produced by the German strains, were isolated and purified for sequencing. Primer walking sequencing using primers oHM133-139 confirmed that the sequence of the German DNA bands was in concordance with the reference mitogenome of SRZ2. Surprisingly, the DNA bands observed in the Chinese strains contained a 3,356 bp fragment that did not match the SRZ2 reference mitogenome. BLASTn analysis of this unique fragment revealed high percent identity to *S. scitamineum* mtDNA (98.55%) and *U. bromivora* mtDNA (96.86%). The updated sequences of the Chinese strains corroborated what was previously observed, with the primer pairs oHM114/115 and oHM119/120 generating amplicons 4,252 bp and 5,889 bp in size, respectively. Accordingly, these updated amplicon sizes explain the larger bands obtained in the PCR experiments (Supplementary Figures S3A, S3B).

**FIGURE 5:**
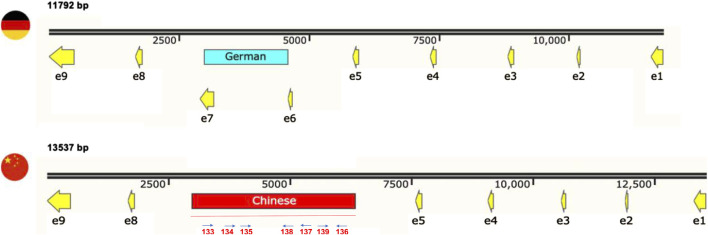
The primer walking sequencing approach previously described revealed unique sequences in *cox1*, with a 1,611 bp fragment in German strains matching SRZ2 mtDNA (black, red, and yellow) and a 3,356 bp fragment in Chinese strains matching *S. scitamineum* and *Ustilago bromivora* (red). The updated gene sizes are indicated in bold. Exons (“e”) are indicated as yellow arrows; the position of primers used in Primer Walking sequence analysis of the polymorphic region in the Chinese isolates is illustrated below the map (Seq Primers oHM133-139).

In the polymorphic region detected in *cox1*, there is an obvious size difference between the German and Chinese versions of this gene (∼1700 bp, Figure 5). Accordingly, the updated mitogenome sizes of Chinese strains are 91,896 bp for SRZCXI2 and SRZCXII2, and 92,158 bp for SRZCXI3. According to the annotated mitogenome of SRZ2, the predicted 528 amino acid polypeptide from *cox1* is encoded by 9 exons. In Chinese strains, the unique sequence overlaps with exons 6 and 7 of the German *cox1* version. Bioinformatic analysis of the unique region in the Chinese version of *cox1* was performed in reference to the *U. bromivora* mitogenome (NCBI Accession No. LT558140.1), as the *S. scitamineum* mitogenome is not fully annotated. BLASTn analysis revealed 5 regions of the unique sequence in the Chinese version of *cox1* with high percent identity to the mitogenome of *U. bromivora*. These regions encompassed two intron-encoded LAGLIDAADG endonucleases flanking three exons of the *U. bromivora cox1*. Additionally, the complete amino acid sequences for *cox1* in Chinese *S. reilianum*, *U. bromivora* and *U. maydis* were compared by MUSCLE analysis, revealing high conservation in all three species (Supplementary Figure S4), suggesting that the putative exons identified in the unique region of *cox1* may, in fact, be part of a fully functional polypeptide that more closely resembles the *U. bromivora cox1* protein.

To verify that the polymorphic region detected in the Chinese isolates was transcribed, total RNA was isolated from the German SRZ2 isolate and from the three Chinese isolates (SRZCXI2, SRZCXI3, and SRZCXII2), cDNA was synthesized from each, and PCR as carried out using primer pair oHM120 and oHM161, that binds in Exons 5 and 9, respectively. The resulting fragments (approximately 770 bp from SRZ2; Supplementary Figure S5) were purified from agarose after gel electrophoresis and sequenced. The sequences showed a high degree of identity among the three Chinese isolates and SRZ2, although some amino acid differences were observed (Supplementary Figure S6)

### 3.4 The *cox1* polymorphism detected in Chinese strains is genetically traceable throughout the fungal life cycle

The life cycle of SRZ is similar to that of the closely related species, *U. maydis*, mainly diverging in the pathogenic profile caused on the plant host, maize. Unlike *U. maydis*, SRZ causes systemic infection, leading to the emergence of teliospore sori in fully mature male and female inflorescences. The dimorphic lifestyle of SRZ is also governed by mating type loci that encode components of a pheromone reaction that allow haploid sporidia to fuse, check further compatibility, and subsequently, enter a sexual lifestyle requiring colonization and invasion of the plant host. Importantly, this transition into sexual reproduction also incorporates appropriate segregation of organelles.

The mitochondrial inheritance mechanism previously described in *U. maydis* is directly linked to the sexual compatibility requirements defined by the *a* mating type locus, which can present itself as two different alleles or idiomorphs (a1 and a2). Accordingly, successful mating may only occur between sporidia of opposing *a* mating types. Notably, the a2 idiomorph also includes genes encoding a degradation-mediated uniparental mitochondrial inheritance mechanism that ensures that offspring will be homoplasmic for the a2 mitotype (Fedler et al., 2009; Mahlert et al., 2009). This mechanism has been previously described, albeit in distinct iterations, in higher eukaryotes, rendering mitochondrial inheritance predominantly “maternal.”

SRZ presents an interesting challenge in the field of organelle inheritance during sexual reproduction, as the *a* mating type locus can be found as three alleles/idiomorphs: a1, a2 and a3. Interestingly, the a2 idiomorph of SRZ has homologous sequences to *lga2* and *rga2*, the genes involved in the uniparental mechanism observed in *U. maydis* (Bortfeld et al., 2004). The molecular mechanism governing mitochondrial inheritance in SRZ has not been characterized, however, based on what was found in *U. maydis*, crosses involving an a2 partner would be predicted to result in offspring of the a2 mitotype. More interestingly, what happens in a cross between a1 and a3 partners remains unclear, and one may only deduce that due to the absence of the molecular machinery controlling mitochondrial inheritance, the resulting offspring will be of mixed mitotypes.

**FIGURE 6:**
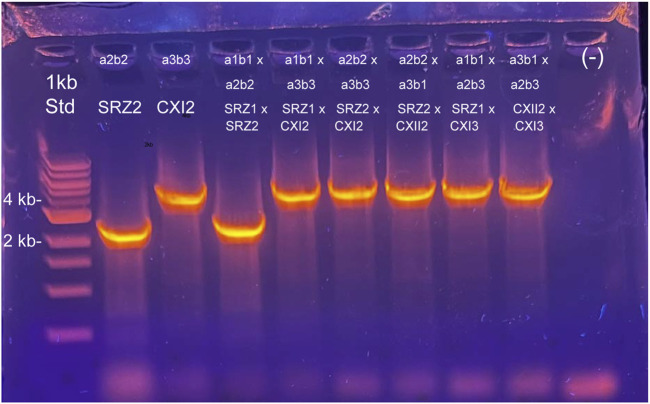
Characterization of mitotypes from teliospore segregants after maize infections. Teliospores were germinated on YPS agar and colonies selected 48–72 h after growth at 28 C; DNA was isolated from re-streaked individual colonies. Four independent colonies from the teliospores germinated for each cross were used for DNA isolation. PCR experiments using primers that work for both German and Chinese strains (114 + 115, 127 + 131) were carried out to determine genetic traceability of the *cox1* polymorphism previously detected. For this figure, agarose gel electrophoresis of PCR products using primers oHM114 and oHM115 are shown, including both control haploid strains (Lanes 2 and 3, SRZ2 and SRZCXI2, respectively), as well as for one segregant colony from each teliospore source: Lane 4, SRZ1 x SRZ2 #1; lane 5, SRZ1 x SRZCXI2 #1; lane 6, SRZ2 x SRZCXI2 #1; lane 7, SRZ2 x SRZCXII2 #1; lane 8, SRZ1 x SRZCXI3 #1; lane 9, SRZCXII2 x SRZCXI3 #1. Lane 1, Quick DNA 1 kb Size standard, New England Biolabs, lane 10, no DNA negative control. Mating types of strains and those in crosses are indicated above each lane; strain names for Chinese isolates are abbreviated CXI2, CXII2, and CXI3. Results for PCR reactions using this primer combination, as well as oHM127/oHM131 on additional independent segregant colonies are shown in Supplementary Figure S8.

The polymorphic region detected in *cox1* provides an auspicious opportunity to test the possible outcomes of different mating scenarios in SRZ. The PCR-based methodology described above represents a method to determine offspring mitotype from specific crosses of SRZ and thereby further dissect the mitochondrial inheritance pattern. As proof-of-principle for this diagnostic tool, primer pairs oHM114/115, oHM127/131 and oHM119/115 were used in the initial screening of mtDNA; accordingly, the mitotypes of all haploid controls tested coincided with their predicted mitotypes (Figure 4C; Figure 6). A second PCR experiment was performed involving primers that work exclusively for German or Chinese mitotypes. Accordingly, the primer pairs oHM115/116 and oHM133/136 were used to detect the Chinese mitotype, producing bands 4,154 bp and 3,115 bp in size, respectively. The primer pairs oHM112/128 were used to detect the German mitotype, producing bands 1,347 bp in size. As expected, haploid controls yielded the corresponding amplicons based on origin (Figure 6; Supplementary Figure S8).

Next, experiments were conducted using mating mixtures of compatible partners (i.e., SRZ2 (German, a2b2) and SRZ1 (German, a1b1), SRZ2 x SRZCXI2 (Chinese, a3b3), SRZ1 x SRZCXI2, SRZ2 x SRZCXI2, SRZ1 x SRZCXI3, SRZCXII2 x SRZCXI3) to infect maize plants. Preliminary pathogenicity screens revealed no significant differences between the crosses tested (data now shown). Total DNA was then extracted from teliospores and primer pairs specific for regions of *cox1* were used to determine mitotype (not shown). As control, haploid sporidia were again subjected to the same PCR experiments and were confirmed to be of the expected mitotype. We predicted that crosses where one partner bore the a2 locus would preferentially yield progeny of the a2 mitotype. In contrast, crosses where the a2 partner was absent would be predicted to follow a biparental inheritance pattern, thus resulting in the amplification of both bands within the same PCR reaction.

### 3.5 PCR screening of *cox1* polymorphic region after infections

Mitotype screening of teliospores resulting from crosses between German and Chinese strains proved difficult, as technical problems with DNA purification from spores often was not of sufficient quality for PCR. Ultimately, an optimized protocol for DNA isolation from teliospores was developed that employed better purified teliospores. Nevertheless, it was still difficult to reproducibly yield PCR products from teliospore-derived DNAs that targeted mitochondrial genomic regions. We hypothesized this might be due to a reduced mitochondrial content in teliospores. To test this hypothesis, quantitative real-time PCR (q-PCR) was conducted on total DNA derived from either sporidia of SRZ2 or SRZCXI2, compared with those derived from teliospores from maize plants infected with a variety of crosses. Consistent with this hypothesis, we found that the ratio of PCR amplicon of a mitochondrial target (conserved regions of the *cox1* gene, amplified with either the combination of primers oHM164 and Ex5Ex8F or NearEx7F and NearEx7R) to that of a nuclear target (*sad1*, sr10077; Ghareeb et al., 2015) was significantly higher for the sporidial samples than for the samples from teliospores (Supplementary Figure S7). Thus, we germinated teliospore samples from each of the infection crosses and subsequently isolated total DNA from representative colonies from such germinated spores. Each independent colony was then used for PCR analysis to test the diagnostic primer combinations (oHM114/115 or oHM127/131). From these analyses we found that crosses involving only one mitotype (i.e., SRZ1 x SRZ2 or SRZCXII2 x SRZCXI3) yielded offspring with only the original starting mitotype (Figure 6; lanes 4 and 9; Supplementary Figure S8A, lanes 4-6; 8C, lanes 1-4; 8D, second panel, SRZ1 x SRZ2 #2 and #4; 8F, CXII2 x CXI3). While the oHM114/115 primer combination indicated a single mitotype for the selected segregant colonies (Figure 6; Supplementary Figures S8A–C), the primer combination oHM127/131 suggested rarely, different mitotypes for different segregant colonies of the same cross (i.e., Supplementary Figure S8E, lane 2 vs. 3; 8E last two lanes vs. Figure 6, lane 7). Moreover, one example (Supplementary Figure S8E, SRZ2 x SRZCXII2#2) shows 2 bands, consistent with heteroplasmy, while another suggests heteroplasmy, but with more Chinese mitotype present in the same individual (Supplementary Figure S8E, SRZ2 x CXII2#3).

## 4 Discussion

The fungus *S. reilianum* provides the ideal setting from which to study mitochondrial inheritance. The degradation-mediated Lga2/Rga2 system first identified and characterized in the related species, *U. maydis*, leads to attractive questions regarding preference of one mitochondrion over the other, possibly emphasizing genomic conflicts and mitochondrial disorders associated with heteroplasmy and DNA incompatibilities. The studies previously carried out in *U. maydis* were solely based on data generated by restriction fragment length polymorphism (RFLP) analysis regarding a polymorphic region in the LSU rRNA gene of the mitochondrial genome (Fedler et al., 2009). Contrastingly, the present study utilized WGS data for the identification of potential polymorphisms among entire mtDNA molecules. The SRZ strains included in this study were deliberately chosen due to their isolated geographic origins (German or Chinese), allowing for identification of distinctive diagnostic polymorphisms. In a natural setting, the SRZ samples used in this study should never have encountered one another and consequently, their mtDNA populations would not have been subject to the bottleneck effect introduced by selective mitochondrial inheritance mechanisms or incestuous propagation in the generation of laboratory strains.

Analysis of the mitogenomic data was done by gene synteny analysis, based on a previous study that compared fungal mitogenomes among different families of fungi (Aguileta et al., 2014). Considering that this study found greater gene order variations among mitogenomes of different species, it was not surprising that the concatenated alignments of the fourteen classical mitochondrial genes of the samples sequenced in this study were identical. Nevertheless, additional multiple sequence alignments identified a variety of polymorphisms intraspecifically. The identification and subsequent verification of these polymorphisms within different regions of the mitochondrial genes served as quality control for the WGS approach, as some of these mutations occurred within protein-coding regions that can be expected to have serious effects on their predicted polypeptide sequences. These mutations needed to be verified individually, as their presence might have implied dysfunctional electron transport chain components.

Some of the polymorphisms detected in this study occur in protein-encoding regions of the mtDNA. Thus, the consequences on the corresponding predicted polypeptide structure and function should be subject to further scrutiny. Notably, the SNP detected in *nad6*, a gene with no introns, results in a shorter predicted polypeptide of one of the subunits of the mitochondrial NADH dehydrogenase. Disruption of the structure and function of this crucial component of the mitochondrial electron transport chain could be detrimental for cell survival, thus increasing the need to confirm this type of mutation. The bioinformatic analysis of this mutation revealed a truncated polypeptide 220 amino acids long, while the predicted sequence should be 226 amino acids in length according to the annotated SRZ2 mitogenome. When compared against the *nad6* protein sequences of related species, it is evident that the mutation truncates the polypeptide at the C-terminus, where it is missing several amino acids shared across all species analyzed. Thus, the *nad6* deletion has the potential to alter the function of its predicted polypeptide, although further experimentation is needed to confirm this *in vitro*. Alternatively, this defect could be offset by the presence of alternative components of the mitochondrial electron transport chain that effectively reroute the movement of electrons through non-canonical pathways to maintain mitochondrial bioenergetics (Juarez et al., 2004; Juarez et al., 2006; Magnani et al., 2008; Lin et al., 2019; Luevano-Martinez et al., 2019).

Further investigation of predicted polymorphisms for other regions of the respective mitogenomes resulted in the discovery of a distinct region of mtDNA among the Chinese samples that was initially undetected by WGS, likely due to the reliance on the reference mitogenome used, SRZ2, which also has German origins. This initial observation implied loss of protein-coding elements of *cox1*, which encodes one of the subunits of complex IV, an important component of the mitochondrial electron transport chain. As revealed by primer walking sequencing, the massive deletion initially detected by WGS in the Chinese samples was actually not a deletion, but rather an insertion of novel DNA that was undetected due to discordance with the annotated SRZ2 genome. BLASTn analysis of the Chinese strains’ *cox1* sequence revealed high similarities to mtDNA of several related species, including *S. scitamineum*, *U. bromivora*, *Melanopsichium pennsylvanicum* and *U. maydis*, but not SRZ2. This finding generates additional questions regarding the origin of novel mtDNA sequence in the Chinese samples. A possible explanation for this phenomenon lies in mtDNA recombination, which has been extensively reported in a variety of fungal systems (Fedler et al., 2009; Aguileta et al., 2014; Fritsch et al., 2014; Wang et al., 2017; Zhang et al., 2020). However, this hypothesis would require Chinese samples of SRZ to have mated (at least abortively) with the related species from which the novel DNA was acquired from (*i.e*., *S. scitamineum*, *U. bromivora*, or *U. maydis*). Interspecific mating has been previously reported among the Ustilaginales, however, few published data have been generated regarding the viability of the potential hybrid offspring (Storfie and Saville, 2021) or the mitochondrial inheritance dynamics that may be involved. Nevertheless, it is possible that even aborted mating between species might lead to exchange of mitochondrial material between the transient partners.

Additional examination of the unique sequence discovered in the Chinese version of *cox1* involved BLASTn analysis against the *U. bromivora* mitochondrial genome. The analysis revealed 5 regions with high percent identity (>86%) that overlapped with intron-encoded LAGLIDAADG endonucleases, which are a group of homing endonucleases (HEs). These DNA elements behave as opportunistic selfish elements usually found in self-splicing introns that recognize site-specific DNA targets to facilitate intron insertion (Megarioti and Kouvelis, 2020). Intron homing has been extensively studied in *Saccharomyces cerevisiae*, with reported efficiencies close to 100% (Lazowska et al., 1994; Eskes et al., 2000; Dickson et al., 2001). However, the relevance of such mobile elements in sexual eukaryotes that have uniparental mitochondrial inheritance mechanisms that potentially purge these selfish elements remains unclear. A recent study in *U. maydis* accidentally discovered the insertion of a mitochondrial HE-encoding gene in the telomeric region of the nuclear genome that is absent from the mitogenome, but resembles one found in the mitogenome of *S. reilianum* (Dutheil et al., 2020). The insertion caused the loss of the homing activity of the selfish gene, as well as the alteration of the function of the protein encoded in the insertion site, which would have led to its eventual elimination by natural selection. This astonishing discovery highlights the power of selfish elements like HEs to influence genetic diversity by restructuring both nuclear and mitochondrial genomes.

The novel DNA sequence acquired by the Chinese SRZ strains may be the result of a combination of the presence of HEs in the mtDNA and potential horizontal gene transfer with other related species like *U. bromivora*, although such an explanation has not yet been explored and requires additional experimental investigation. However, horizontal gene transfer has been extensively reported in plants (Vaughn et al., 1995; Bergthorsson et al., 2003; Quispe-Huamanquispe et al., 2017) and, more recently, in *in vitro* co-cultures of human cells and, incredibly, in *in vivo* mouse tumor models (Berridge et al., 2016). The region analyzed also included 3 exons of the *U. bromivora cox1*. Interestingly, the coding sequence of *cox1* in *U. bromivora* is made up of 14 exons, in contrast to the 9 exons that make up the *S. reilianum cox1*. Nevertheless, the resulting polypeptides are similar in size (524 and 528 amino acids, respectively). Accordingly, the exons present in the unique Chinese region of *cox1* may make up for the loss of the exons proper of the German version.

MUSCLE analysis of the amino acid sequences of Cox1 revealed high identity among *S. reilianum*, *U. bromivora* and *U. maydis*. Interestingly, the amino acid sequence of the *S. reilianum* Cox1 protein is more similar to that of *U. maydis*, both resulting in proteins 528 amino acids in length. Together, these last bioinformatic findings substantiate the hypothesis that the unique sequence in the Chinese version of *cox1* in *S. reilianum* contains exons highly similar to those found in *U. bromivora*, likely leading to production of a fully functional polypeptide. Additionally, the analyzed strains in this study do not differ in growth or survival rates under standard laboratory conditions; thus, it can be assumed that this mutation may not have deleterious effects on the predicted polypeptide. Nevertheless, specific respiratory studies in SRZ are required to confirm normal biochemical function of the mitochondrial electron transport chain, specifically looking at complex IV activity.

In the present study, we successfully developed the primer combination oHM114/115 as a diagnostic tool to detect unique German and Chinese amplicons (primers bind in the immediate vicinities of the *cox1* polymorphic region) for all starting strains. Additional primer combinations could similarly be used to successfully detect either German or Chinese mitotypes specifically.

The pathogenicity assays performed in this study served the immediate purpose of producing teliospores from different crosses between German and Chinese strains. Total DNA isolation was performed on all teliospore samples and specific mtDNA primers were used to verify integrity of the extracted nucleic acids and PCR suitability. Mitotype screening of the teliospores produced in the pathogenicity assays was based on the polymorphic region detected in *cox1*. Initially, we faced technical hurdles, first with quality of DNA isolation from teliospore samples and later, with reproducibility for PCR from mitochondrial DNA targets from such total DNA samples. While nuclear targets, e.g., sr10077 (the *sad1* gene (Ghareeb et al., 2015)) were reproducibly amplified, primer combinations targeting various regions, both within the polymorphic *cox1* gene, and for other targets (i.e., *nad6*, not shown) failed to yield reproducible results. Suspecting this irreproducibility might be due to low mitochondrial content in teliospores, this hypothesis was tested using quantitative PCR (qPCR) to assess relative amounts of genomic (nuclear) DNA vs. mitochondrial DNA in sporidial samples compared with teliospore samples. Consistent with this hypothesis, the ratio of mitochondrial vs. nuclear target for sporidial compared with teliospore samples was significantly higher for the sporidial haploid strains (i.e., both SRZ2 and SRZCXI2) than for any of the teliospore samples from all crosses used for maize infection. Moreover, the relative amount of mitochondrial DNA appeared to be 2-to-2.5-fold higher in the SRZCXI2 (Chinese) strain compared to the SRZ2 (German) strain (Supplementary Figure S7). The results of subsequent PCR screening of segregants of germinated teliospores from crosses (Figure 6; Supplementary Figure S8), showed that the primer sets used, which should differentially amplify either the German or Chinese strains, successfully distinguished not only the control haploid reference strains, but also produced consistent results for the crosses involving parents with the same starting mitotype. (i.e., either both German or both Chinese parental strains; Figure 6, lanes 4 and 9; Supplementary Figure S8A, lanes 4-6; 8C, lanes 1-4; 8D, last two lanes of second panel; 8F, CXII2 x CXI3 #1-#4). Crosses between parents of different mitotypes (i.e., German vs. Chinese) mostly appeared to show the Chinese mitotype regardless of the mating-type of the Chinese partner in the mating; rarely the results indicated different mitotypes for different segregants of the same cross or even clear heteroplasmy from the same cross. One possible explanation for this latter observation, is the increased amount of mitochondrial DNA (and likely mitochondria) observed in at least one Chinese isolate, SRZCXI2, compared to the German isolate, SRZ2. This may also explain situations where there appears to be biparental inheritance for a given cross, but the predominant mitotype is that of the Chinese strain. Thus, counter to our original hypothesis, the parental strains bearing the a2 mating-type containing the orthologues of *lga2* and *rga2* of *U. maydis*, did not apparently control mating-type of the offspring from crosses, at least not between the German and Chinese isolates. Future experiments to investigate whether the *lga2* and *rga2* orthologues play any roles in mitochondrial inheritance could include the development of transgenic German and Chinese strains in which the respective a2 loci are exchanged between the prospective mating partners and then mitotype is again followed in the segregants of germinated teliospores. Such experiments will aid in the further characterization of mitochondrial dynamics and control of inheritance in *S. reilianum*.

## Data Availability

The datasets presented in this study can be found in online repositories. The names of the repository/repositories and accession number(s) can be found below: https://www.ncbi.nlm.nih.gov/, BioProject ID PRJNA992771.

## Supplementary material

SUPPLEMENTARY FIGURE S1 Map showing *cox1* gene (positions 43,538 … 60,477) from strain SRZ2, with primer locations indicated. Additionally, primer combinations oHM120 and oHM161 produce an approximately 770 bp fragment from cDNA of the respective strains.

SUPPLEMENTARY FIGURE S2 Electropherograms and alignment of *nad6* from SRZ strains with the SRZ2 reference genome reveals a point mutation in all strains, including SRZ2. Representative electropherograms of SRZ2 and SRZCXI2 *nad6* genes, from positions 638 to 681 (and reverse strand sequence) compared to the corresponding sequence of the SRZ2 reference genome (NCBI Accession No. GCA_000230245.1). The single letter abbreviations for the codons are indicate above the reference sequence. The 642G, present in the reference genome (highlighted in red) is missing in all the corresponding *nad6* genes examined in this study, indicated here by the red arrows, for both forward (SRZ2For, SRZCXI2For) and reverse sequences (SRZ2Rev, SRZCXI2Rev).

SUPPLEMENTARY FIGURE S3 Diagnostic PCR experiments were carried out to confirm the *cox1* polymorphism detected using the primer combinations **(A)** oHM114/115 (expected = 2,507 bp in German or 1,158 bp in Chinese) and **(B)** oHM119/120 (expected = 4,143 bp in German and 2,795 bp in Chinese strains).

SUPPLEMENTARY FIGURE S4 MUSCLE analysis of predicted polypeptide from sequenced Chinese *S. reilianum cox1* against known polypeptides (*U. bromivora* = Ub, *U. maydis* = Um). Amino acids with similar physico-chemical properties are represented by the same color. Bars represent amino acid conservation across all sequences analyzed, with darker and taller bars corresponding to higher conservation indexes. Amino acids in bold make up the consensus sequence and represent 100% conservation in that position.

SUPPLEMENTARY FIGURE S5 Analysis of the transcribed region spanning Exon5-Exon9. Total RNAs were isolated from SRZ strains grown on PDA for 2 days at 28°C as described in the Materials and Methods. cDNAs were synthesized. PCR reactions using primers oHM120 and oHM161 were conducted using either cDNA (lanes 1-4) or original RNA samples without cDNA synthesis (lands 5-8) as a negative control to assure no mtDNA contamination. PCR reactions were analyzed via electrophoresis in agarose gels. Lanes 1-4 were from SRZ2, SRZCXI2, SRZCXI3, and SRZCXII2, respectively; lanes 5-8 were from SRZ2, SRZCXI2, SRZCXI3, and SRZCXII2, respectively; lane 10 was the Quick DNA 1 kb Size standard, New England Biolabs.

SUPPLEMENTARY FIGURE S6 ClustalW alignments of nucleotide and predicted amino acid sequences from the purified cDNAs in supplementary figure S5. Identified nucleotide difference in SRZ2 relative to the other isolates are indicated in larger font and colored orange.

SUPPLEMENTARY FIGURE S7 Relative quantification of mitochondrial DNA of teliospores from a cross of SRZ1 with SRZCXI2 used to infect maize, and also, from an infection cross of SRZ1 with SRZ2. **(A)** Using SRZ2CXI2 as a reference sample; **(B)** Using SRZ2 as a reference sample. For quantitative PCR (qPCR) mitochondrial targets of the *cox1* gene were amplified using primer combinations NearEx7F/NearEx7R or oHM164/Ex5Ex8F and amount of mt genomic amplicon was normalized relative to the corresponding nuclear-encoded gene amplicon [*sad1* (sr10077) Ghareeb et al., 2015]. The data are represented such that, when SRZCXI2 was used as the reference sample **(A)**, its normalized amplification is set to 1.0 and the amount of amplicon for the other samples is shown relative to that; similarly, in **(B)**, where SRZ2 is the reference, its normalized amplification of the mitochondrial targets is set to 1.0, to which all other samples are compared.

SUPPLEMENTARY FIGURE S8 Agarose gel electrophoresis illustrating additional PCR screening, using primer oHM114/oHM115 **(A-C)** or oHM127/oHM131 **(D-F)**. PCR reactions for control haploid strains SRZ2 and SRZCXI2 are shown in Panels A and D only. **(A)** Lane 1, Quick DNA 1 kb Size standard, New England Biolabs; lanes 2 and 3, SRZ2 and SRZCXI2 respectively; German strain crosses, lanes 4-6, SRZ1 x SRZ2 #1, #2, and #3, respectively. **(B)** Lane 1, Quick DNA 1 kb Size standard; the remaining labelled lanes show crosses between a strain of German origin and a compatible one of Chinese origin: SRZ2 x SRZCXII2 #3, SRZ2 x SRZCXII2 #1 and SRZ2 x SRZCXII2 #4, respectively. **(C)** Crosses between Chinese strains, lanes 1-4: SRZCXII2 x SRZCXI3 #1, #2, #3, and #4, respectively; lane 5, no DNA negative control; lane 6, Quick DNA 1 kb Size standard. **(D)** first gel panel, Lane 1, Quick DNA 1 kb Size standard; next are shown SRZ2 and SRZCXI2 respectively; then, SRZ1 x SRZ2 #1 and #3; SRZ1 x SRZCXII2 #3; second gel panel, SRZ2 and SRZCXI2, respectively; then, SRZ1 x SRZ2 #2 #4, respectively (samples in the two panels were run in separate gels. **(E)** Lane 1, Quick DNA 1 kb Size standard; lanes 2 and 3, SRZ2 x SRZCXI2 #1 and #2, respectively; last two lanes, SRZ2 x SRZCXII2 #2 and #3, respectively. **(F)** Lane 1, Quick DNA 1 kb Size standard; lane 5, SRZ1 x SRZCXI3 #4; lanes 6-9.SRZCXII2 x SRZCXI3 #1-#4, respectively; last lane, no DNA negative control.
