# Supplementary material for: Comparative mitogenomic analysis of Sporisorium reilianum f. sp. zeae suggests recombination events during its evolutionary history
Source: Front Physiol. 2024 Sep 6;15:1264359. doi: 10.3389/fphys.2024.1264359 (PMC11413489; doi:10.3389/fphys.2024.1264359)
Supplement: Supplementary file 1 [file DataSheet2.pdf]

Supplementary Material

SUPPLEMENTARY FIGURE 1. Map showing *cox1* gene (positions 43,538…60,477) from strain SRZ2, with primer locations indicated. Additionally, primer combinations oHM120 and oHM161 produce an approximately 770 bp fragment from cDNA of the respective strains.

SUPPLEMENTARY FIGURE 2. Electropherograms and alignment of nad6 from SRZ strains with the SRZ2 reference genome reveals a point mutation in all strains, including SRZ2. Representative electropherograms of SRZ2 and SRZCXI2 nad6 genes, from positions 638 to 681 (and reverse strand sequence) compared to the corresponding sequence of the SRZ2 reference genome (NCBI Accession No. GCA_000230245.1). The single letter abbreviations for the codons are indicate above the reference sequence. The 642G, present in the reference genome (highlighted in red) is missing in all the corresponding nad6 genes examined in this study, indicated here by the red arrows, for both forward (SRZ2For, SRZCXI2For) and reverse sequences (SRZ2Rev, SRZCXI2Rev).

SUPPLEMENTARY FIGURE 3. Diagnostic PCR experiments were carried out to confirm the *cox1* polymorphism detected using the primer combinations (A) oHM114/115 (expected = 2507 bp in German or 1158 bp in Chinese) and (B) oHM119/120 (expected = 4143 bp in German and 2795 bp in Chinese strains).

SUPPLEMENTARY FIGURE 4.   MUSCLE analysis of predicted polypeptide from sequenced Chinese *S. reilianum cox1* against known polypeptides (*U. bromivora* = Ub, *U. maydis* = Um). Amino acids with similar physico-chemical properties are represented by the same color. Bars represent amino acid conservation across all sequences analyzed, with darker and taller bars corresponding to higher conservation indexes. Amino acids in bold make up the consensus sequence and represent 100% conservation in that position.

SUPPLEMENTARY FIGURE 5. Analysis of the transcribed region spanning Exon5-Exon9. Total RNAs were isolated from SRZ strains grown on PDA for 2 days at 28°C as described in the Materials and Methods. cDNAs were synthesized. PCR reactions using primers oHM120 and oHM161 were conducted using either cDNA (lanes 1-4) or original RNA samples without cDNA synthesis (lands 5-8) as a negative control to assure no mtDNA contamination. PCR reactions were analyzed via electrophoresis in agarose gels. Lanes 1-4 were from SRZ2, SRZCXI2, SRZCXI3, and SRZCXII2, respectively; lanes 5-8 were from SRZ2, SRZCXI2, SRZCXI3, and SRZCXII2, respectively; lane 10 was the Quick DNA 1 kb Size standard, New England Biolabs.

SUPPLEMENTARY FIGURE 6. ClustalW alignments of nucleotide and predicted amino acid sequences from the purified cDNAs in Supplementary Figure 5. Identified nucleotide difference in SRZ2 relative to the other isolates are indicated in larger font and colored orange.

SUPPLEMENTARY FIGURE 7. Relative quantification of mitochondrial DNA of teliospores from a cross of SRZ1 with SRZCXI2 used to infect maize, and also, from an infection cross of SRZ1 with SRZ2. (A) Using SRZ2CXI2 as a reference sample; (B) Using SRZ2 as a reference sample. For quantitative PCR (qPCR) mitochondrial targets of the *cox1* gene were amplified using primer combinations NearEx7F/NearEx7R or oHM164/Ex5Ex8F and amount of mt genomic amplicon was normalized relative to the corresponding nuclear-encoded gene amplicon (sad1 (sr10077) Ghareeb et al., 2015)). The data are represented such that, when SRZCXI2 was used as the reference sample (A), its normalized amplification is set to 1.0 and the amount of amplicon for the other samples is shown relative to that; similarly, in (B), where SRZ2 is the reference, its normalized amplification of the mitochondrial targets is set to 1.0, to which all other samples are compared.

SUPPLEMENTARY FIGURE 8. Agarose gel electrophoresis illustrating additional PCR screening, using primer oHM114/oHM115 (A-C) or oHM127/oHM131 (D-F). PCR reactions for control haploid strains SRZ2 and SRZCXI2 are shown in Panels A and D only. (A) Lane 1, Quick DNA 1 kb Size standard, New England Biolabs; lanes 2 and 3, SRZ2 and SRZCXI2 respectively; German strain crosses, lanes 4-6, SRZ1 x SRZ2 #1, #2, and #3, respectively. (B) Lane 1, Quick DNA 1 kb Size standard; the remaining labelled lanes show crosses between a strain of German origin and a compatible one of Chinese origin: SRZ2 x SRZCXII2 #3, SRZ2 x SRZCXII2 #1 and SRZ2 x SRZCXII2 #4, respectively. (C) Crosses between Chinese strains, lanes 1-4: SRZCXII2 x SRZCXI3 #1, #2, #3, and #4, respectively; lane 5, no DNA negative control; lane 6, Quick DNA 1 kb Size standard. (D) first gel panel, Lane 1, Quick DNA 1 kb Size standard; next are shown SRZ2 and SRZCXI2 respectively; then, SRZ1 x SRZ2 #1 and #3; SRZ1 x SRZCXII2 #3; second gel panel, SRZ2 and SRZCXI2, respectively; then, SRZ1 x SRZ2 #2 #4, respectively (samples in the two panels were run in separate gels. (E) Lane 1, Quick DNA 1 kb Size standard; lanes 2 and 3, SRZ2 x SRZCXI2 #1 and #2, respectively; last two lanes, SRZ2 x SRZCXII2 #2 and #3, respectively. (F) Lane 1, Quick DNA 1 kb Size standard; lane 5, SRZ1 x SRZCXI3 #4; lanes 6-9.SRZCXII2 x SRZCXI3 #1-#4, respectively; last lane, no DNA negative control.
