# Supplementary figures and images for: Comparative mitogenomic analysis of Sporisorium reilianum f. sp. zeae suggests recombination events during its evolutionary history
Source: Front Physiol. 2024 Sep 6;15:1264359. doi: 10.3389/fphys.2024.1264359 (PMC11413489; doi:10.3389/fphys.2024.1264359)

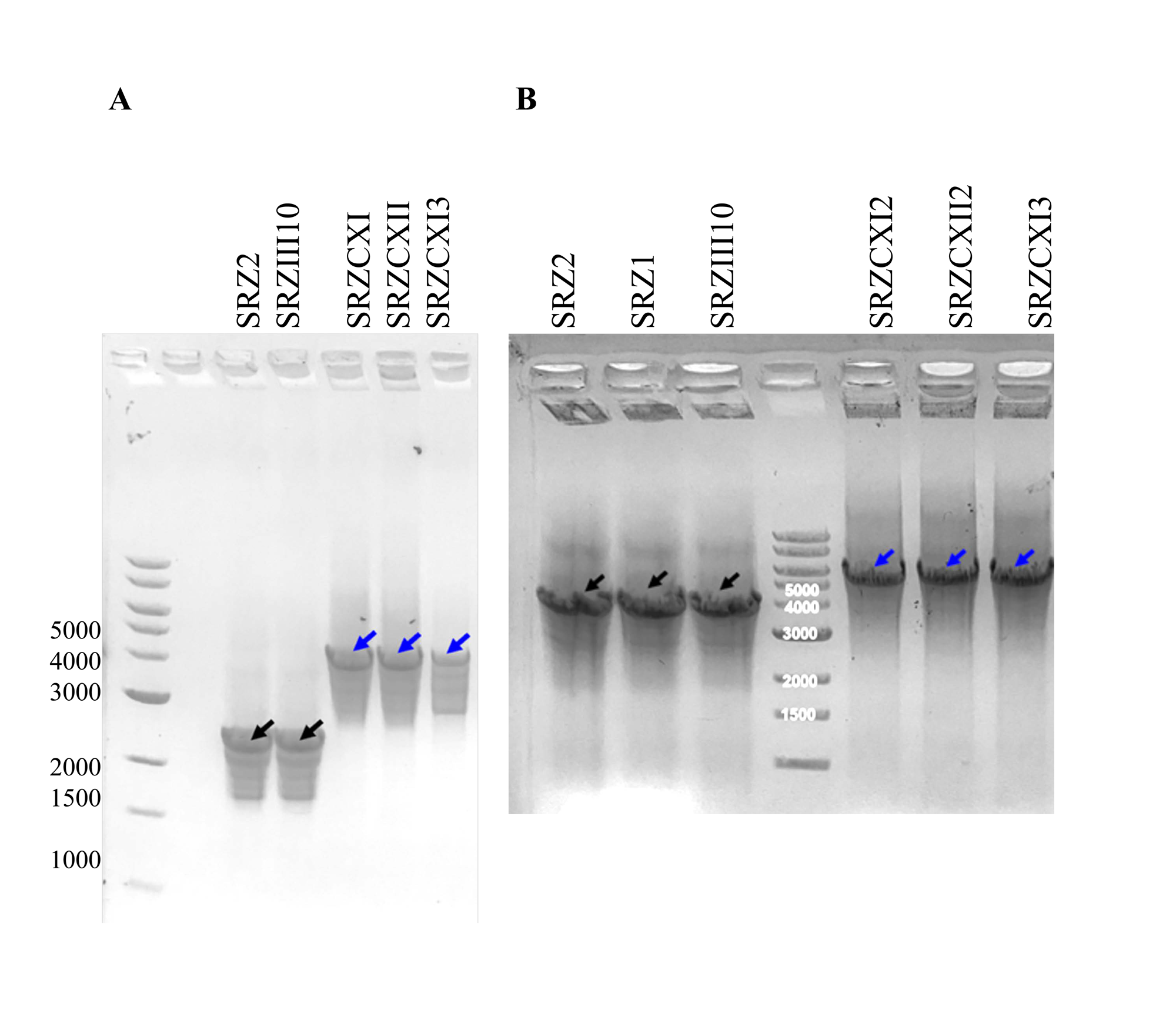

Supplement: Supplementary file 2 [file Image3.tif]

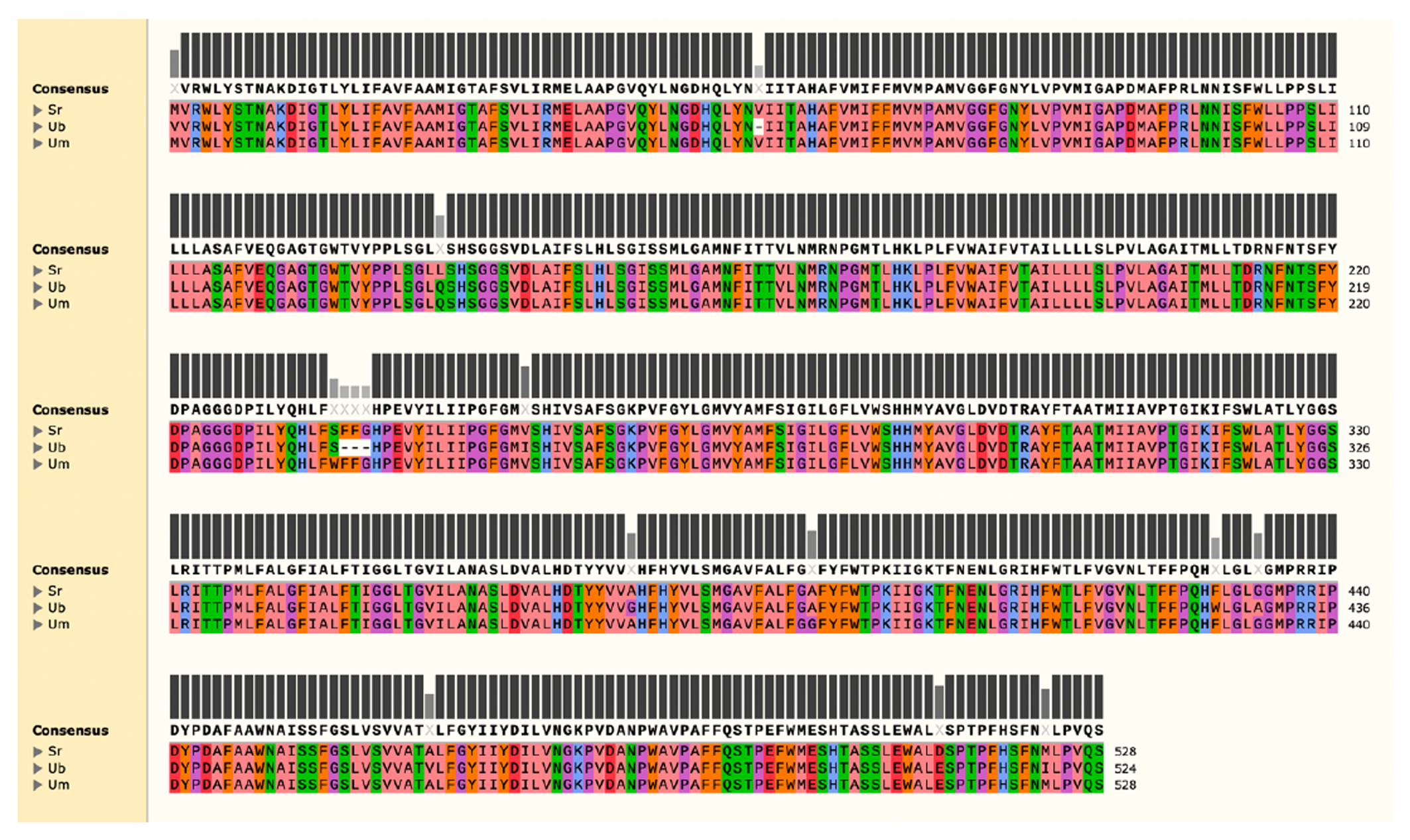

Supplement: Supplementary file 3 [file Image4.tif]

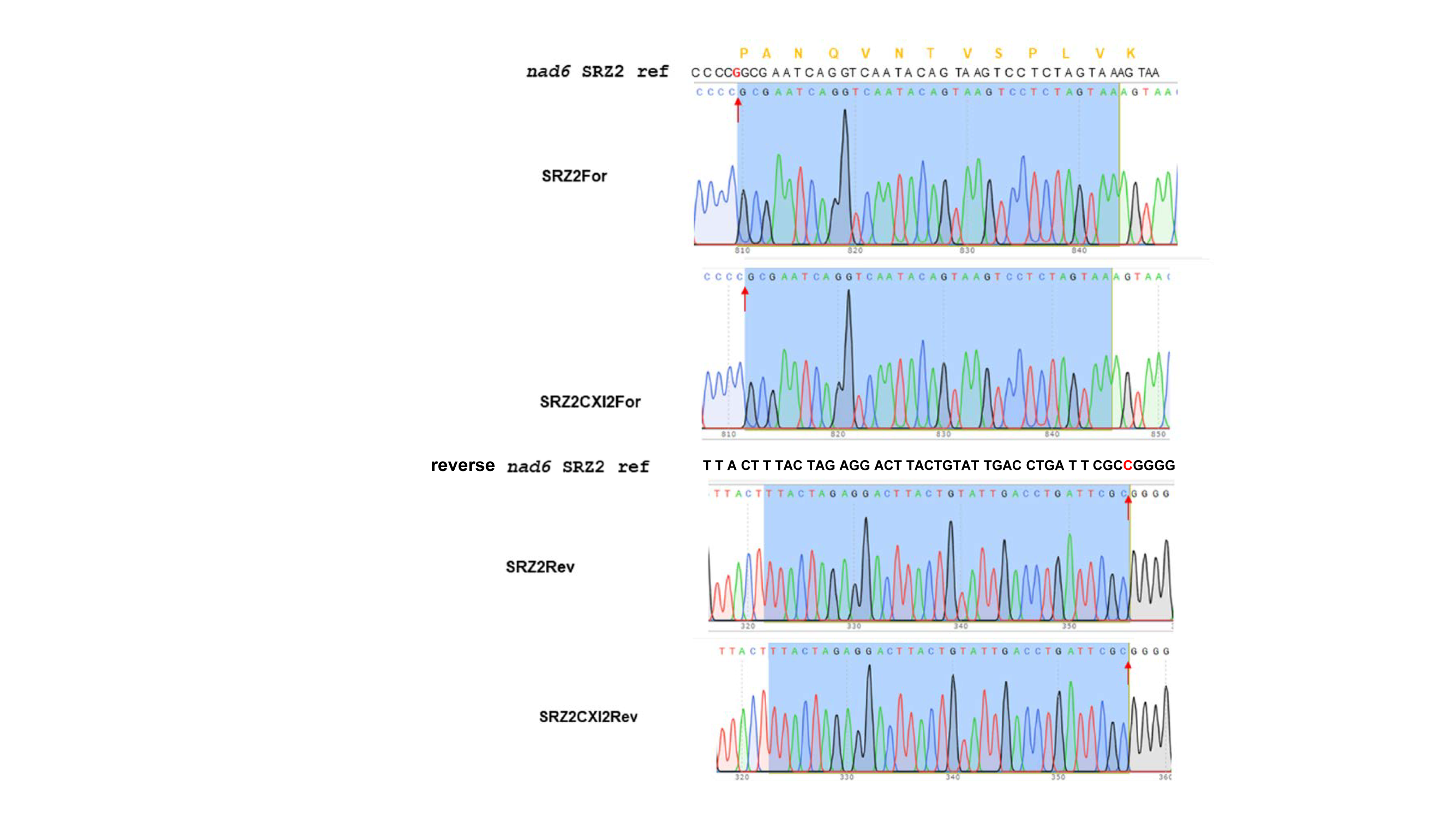

Supplement: Supplementary file 5 [file Image2.tif]

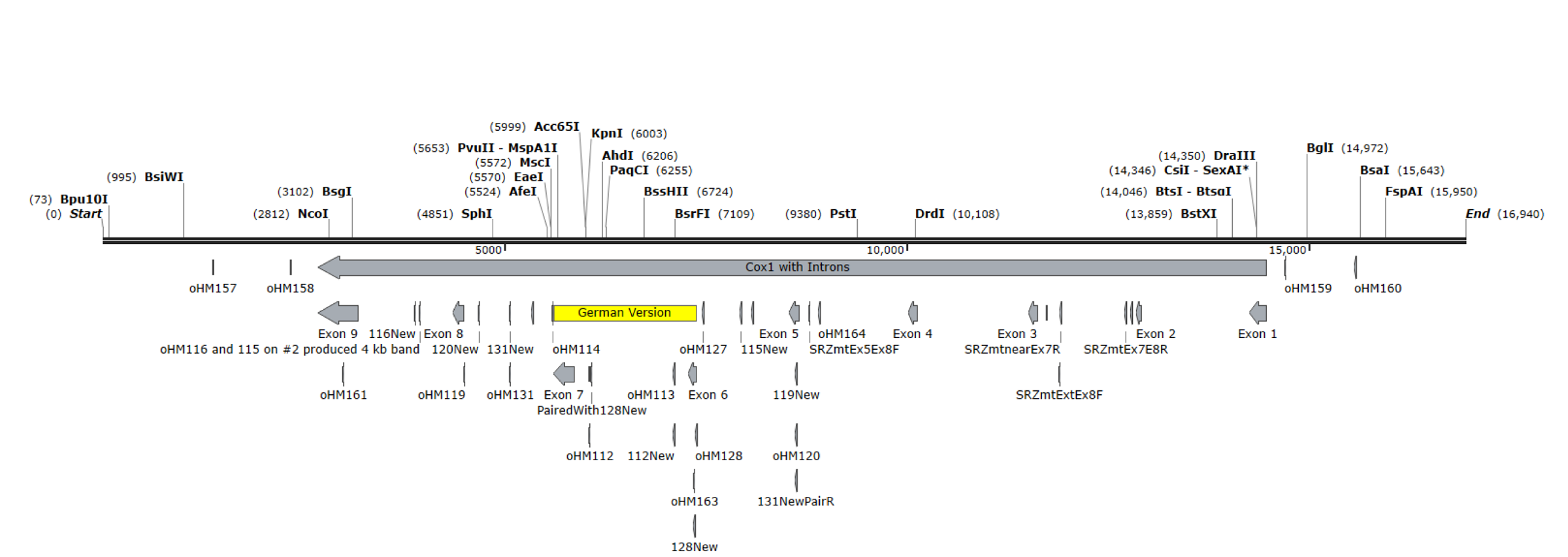

Supplement: Supplementary file 6 [file Image1.tif]

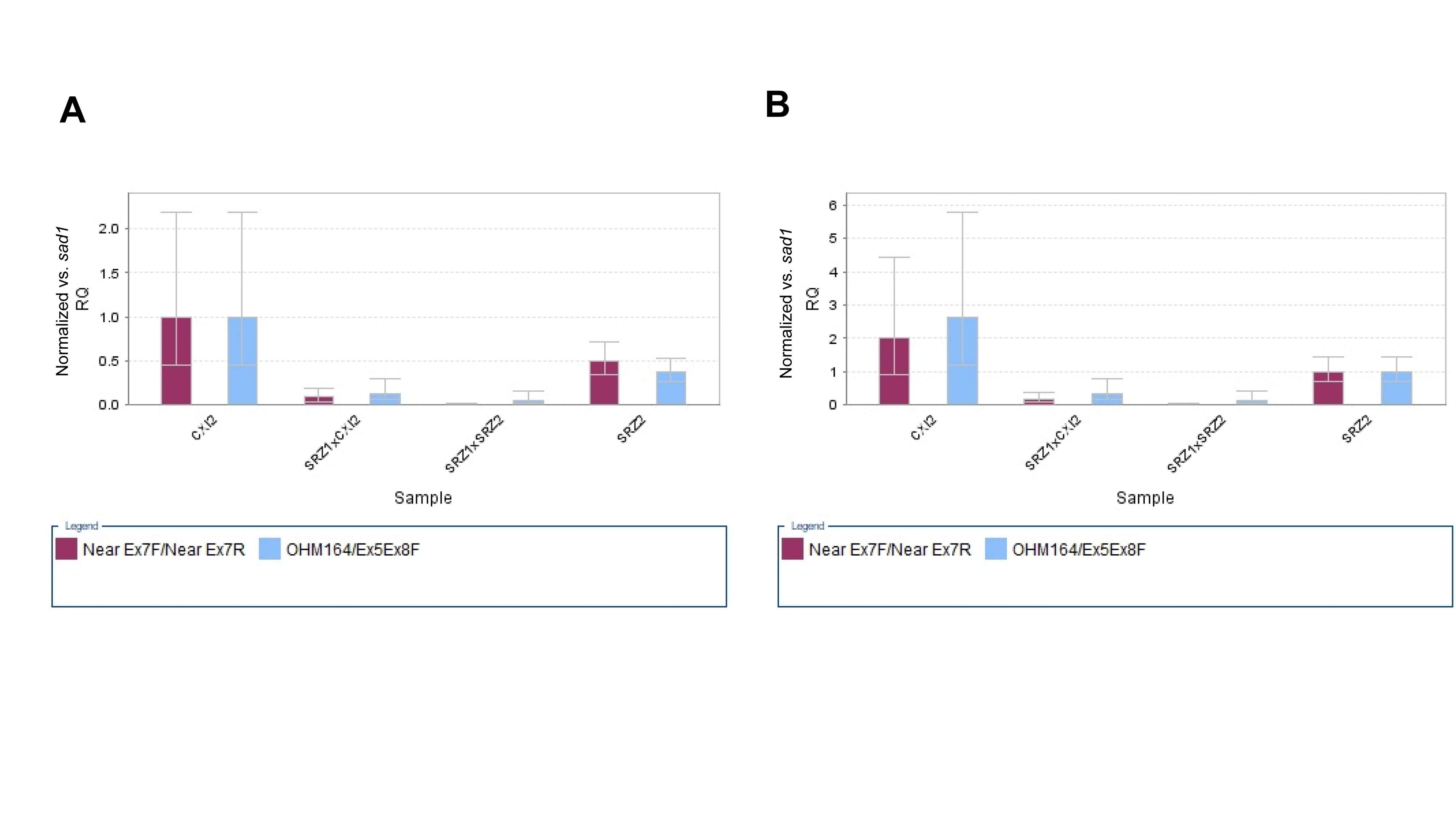

Supplement: Supplementary file 7 [file Image7.tif]

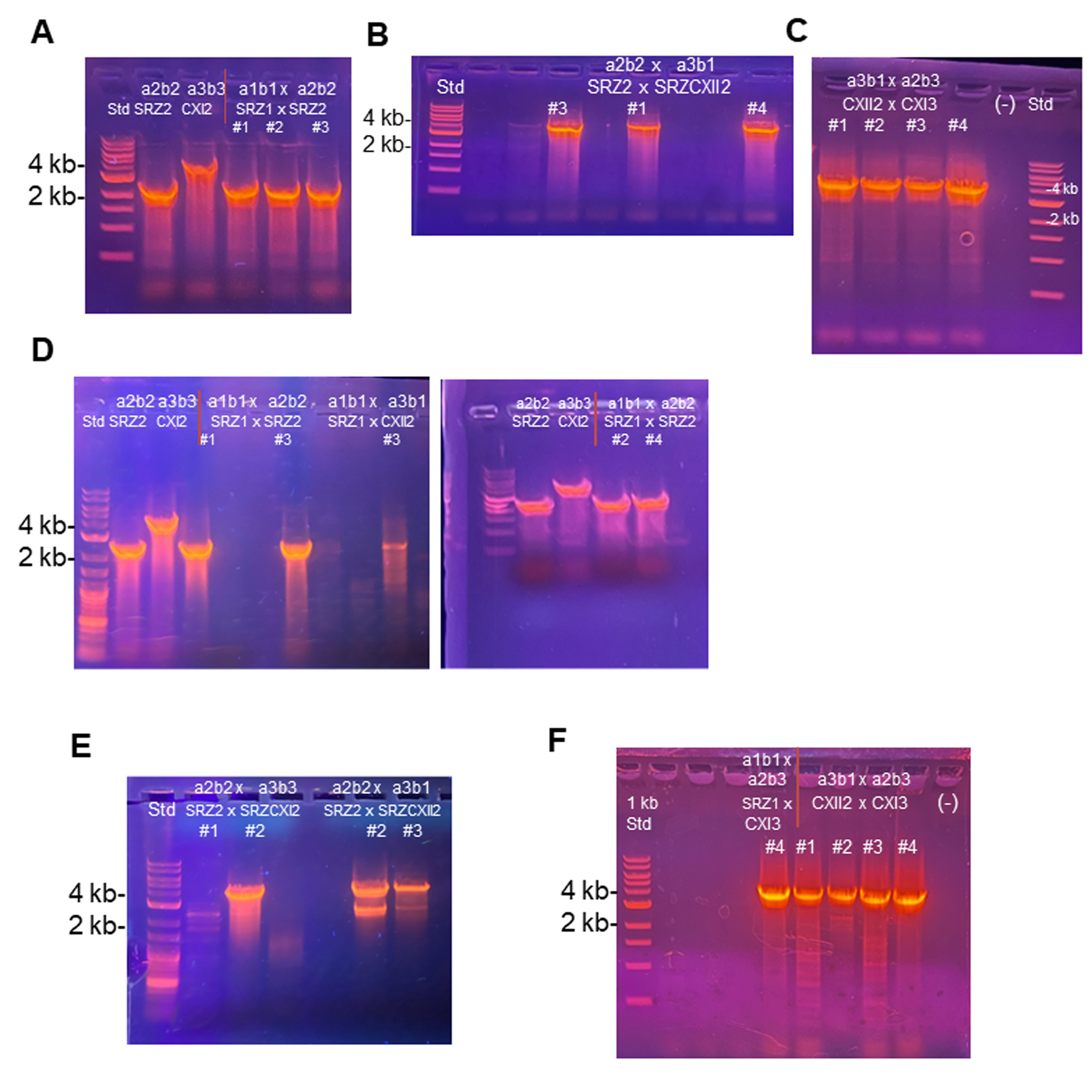

Supplement: Supplementary file 9 [file Image8.tif]

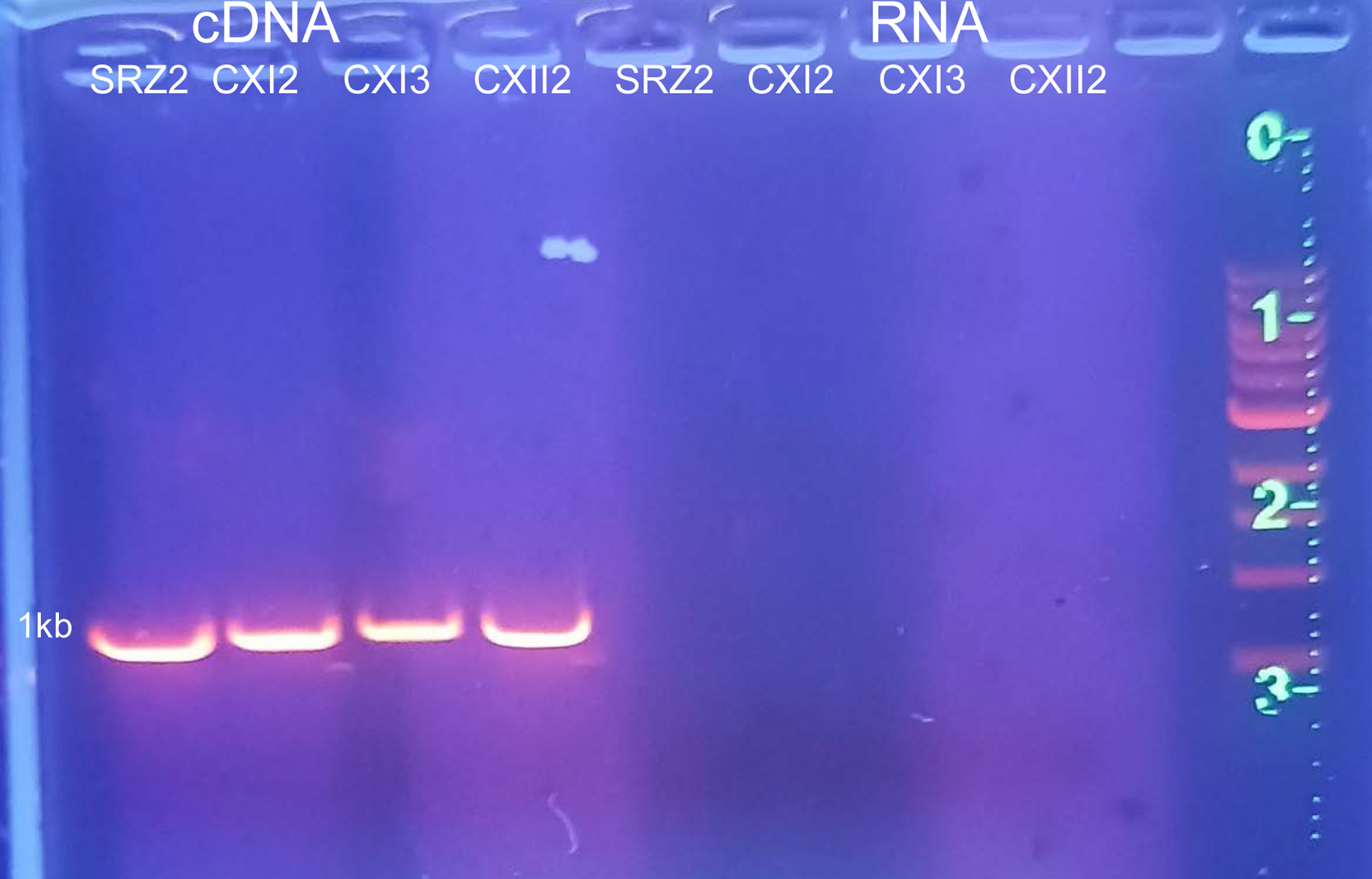

Supplement: Supplementary file 10 [file Image5.tif]
