# Supplementary material for: Comparative mitogenomic analysis of Sporisorium reilianum f. sp. zeae suggests recombination events during its evolutionary history
Source: Front Physiol. 2024 Sep 6;15:1264359. doi: 10.3389/fphys.2024.1264359 (PMC11413489; doi:10.3389/fphys.2024.1264359)
Supplement: Supplementary file 4 [file Image6.pdf]

CLUSTAL O(1.2.4) multiple sequence alignment

```
XI2Ex5-Ex9      ---------------------CGAAACCCACAGATGACACTACATAAACTACCACTATTT      39
XI3Ex5-Ex9      -----------------------AACCCCCCGGATGACACTACATAAACTACCACTATTT      37
SRZ2Ex5-9       ATTACTACAGTTCTTAACATGCGAAACCCAGGAATGACACTACATAAACTACCACTATTT      60
XII2Ex5-Ex9     --------------------------------GATGACACTACATAAACTACCACTATTT      28
                                                .***************************

XI2Ex5-Ex9      GTTTGGGCTATCTTTGTTACAGCTATTCTACTGCTACTATCACTACCAGTACTAGCTGGT      99
XI3Ex5-Ex9      GTTTGGGCTATCTTTGTTACAGCTATTCTACTGCTACTATCACTACCAGTACTAGCTGGT      97
SRZ2Ex5-9       GTTTGGGCTATCTTTGTTACAGCTATTCTACTGCTACTATCACTACCAGTACTAGCTGG-      119
XII2Ex5-Ex9     GTTTGGGCTATCTTTGTTACAGCTATTCTACTGCTACTATCACTACCAGTACTAGCTGGT      88
                *********************************************************** 

XI2Ex5-Ex9      GCAATTACAATGCTACTAACAGACCGGAACTTTAATACATCATTCTATGATCCAGCAGGA      159
XI3Ex5-Ex9      GCAATTACAATGCTACTAACAGACCGGAACTTTAATACATCATTCTATGATCCAGCAGGA      157
SRZ2Ex5-9       GCAATTACAATGCTACTAACAGACCGGAACTTTAATACATCATTCTATGATCCAGCAGGA      179
XII2Ex5-Ex9     GCAATTACAATGCTACTAACAGACCGGAACTTTAATACATCATTCTATGATCCAGCAGGA      148
                ************************************************************

XI2Ex5-Ex9      GGTGGAGATCCAATTCTTTACCAACACCTATTCTGGTTCTTTGGTCACCCAGAAGTTTAT      219
XI3Ex5-Ex9      GGTGGAGATCCAATTCTTTACCAACACCTATTCTGGTTCTTTGGTCACCCAGAAGTTTAT      217
SRZ2Ex5-9       GGTGGAGATCCAATTCTTTACCAACACCTATTCTCATTCTTTGGTCACCCAGAAGTTTAT      239
XII2Ex5-Ex9     GGTGGAGATCCAATTCTTTACCAACACCTATTCTGGTTCTTTGGTCACCCAGAAGTTTAT      208
                ********************************** .************************

XI2Ex5-Ex9      ATCCTAATTATCCCTGGATTTGGAATGGTTAGTCACATCGTAAGTGCATTCTCAGGTAAA      279
XI3Ex5-Ex9      ATCCTAATTATCCCTGGATTTGGAATGGTTAGTCACATCGTAAGTGCATTCTCAGGTAAA      277
SRZ2Ex5-9       ATCCTAATTATCCCTGGATTTGGAATGGTTAGTCACATCGTAAGTGCATTCTCAGGTAAA      299
XII2Ex5-Ex9     ATCCTAATTATCCCTGGATTTGGAATGGTTAGTCACATCGTAAGTGCATTCTCAGGTAAA      268
                ************************************************************

XI2Ex5-Ex9      CCAGTATTTGGATACCTAGGAATGGTTTATGCTATGTTCAGTATTGGAATCCTAGGATTC      339
XI3Ex5-Ex9      CCAGTATTTGGATACCTAGGAATGGTTTATGCTATGTTCAGTATTGGAATCCTAGGATTC      337
```

```
SRZ2Ex5-9          CCAGTATTTGGATACCTAGGAATGGTTTATGCTATGTTTAGTATTGGAATTCTAGGATTC   359
XII2Ex5-Ex9        CCAGTATTTGGATACCTAGGAATGGTTTATGCTATGTTCAGTATTGGAATCCTAGGATTC   328
                   ************************************** *********** *********

XI2Ex5-Ex9         CTAGTATGGAGTCACCACATGTATGCTGTAGGACTAGATGTTGATACTCGAGCTTACTTT   399
XI3Ex5-Ex9         CTAGTATGGAGTCACCACATGTATGCTGTAGGACTAGATGTTGATACTCGAGCTTACTTT   397
SRZ2Ex5-9          CTGGTATGGAGTCACCACATGTATGCTGTTGGACTAGATGTTGATACACGAGCTTACTTT   419
XII2Ex5-Ex9        CTAGTATGGAGTCACCACATGTATGCTGTAGGACTAGATGTTGATACTCGAGCTTACTTT   388
                   **.**************************:*****************:************

XI2Ex5-Ex9         ACAGCTGCTACAATGATTATTGCTGTTCCTACAGGAATTAAAATCTTCTCATGGCTAGCT   459
XI3Ex5-Ex9         ACAGCTGCTACAATGATTATTGCTGTTCCTACAGGAATTAAAATCTTCTCATGGCTAGCT   457
SRZ2Ex5-9          ACAGCTGCTACAATGATTATTGCTGTTCCTACAGGAATTAAAATCTTCTCATGGCTTGCT   479
XII2Ex5-Ex9        ACAGCTGCTACAATGATTATTGCTGTTCCTACAGGAATTAAAATCTTCTCATGGCTAGCT   448
                   ********************************************************:***

XI2Ex5-Ex9         ACACTATACGGTGGTTCTCTACGAATCACTACACCTATGCTATTTGCTCTTGGGTTTATT   519
XI3Ex5-Ex9         ACACTATACGGTGGTTCTCTACGAATCACTACACCTATGCTATTTGCTCTTGGGTTTATT   517
SRZ2Ex5-9          ACACTATACGGTGGTTCTCTACGAATCACTACACCTATGCTATTTGCTCTTGGGTTTATT   539
XII2Ex5-Ex9        ACACTATACGGTGGTTCTCTACGAATCACTACACCTATGCTATTTGCTCTTGGGTTTATT   508
                   ************************************************************

XI2Ex5-Ex9         GCTCTATTTACAATTGGAGGTCTAACAGGAGTAATTCTAGCTAATGCTTCACTAGATGTT   579
XI3Ex5-Ex9         GCTCTATTTACAATTGGAGGTCTAACAGGAGTAATTCTAGCTAATGCTTCACTAGATGTT   577
SRZ2Ex5-9          GCTCTATTTACAATTGGAGGTCTAACAGGAGTAATTCTAGCTAATGCTTCACTAGATGTT   599
XII2Ex5-Ex9        GCTCTATTTACAATTGGAGGTCTAACAGGAGTAATTCTAGCTAATGCTTCACTAGATGTT   568
                   ************************************************************

XI2Ex5-Ex9         GCTCTACACGATACATACTACGTTGTTGCTCACTTCCACTATGTTCTATCAATGGGTGCA   639
XI3Ex5-Ex9         GCTCTACACGATACATACTACGTTGTTGCTCACTTCCACTATGTTCTATCAATGGGTGCA   637
SRZ2Ex5-9          GCTCTACACGATACATACTACGTTGTTGCTCACTTCCACTATGTTCTATCAATGGGTGCA   659
XII2Ex5-Ex9        GCTCTACACGATACATACTACGTTGTTGCTCACTTCCACTATGTTCTATCAATGGGTGCA   628
                   ************************************************************

XI2Ex5-Ex9         GTATTTGCTCTATTTGGTGCATTCTACTTCTGGACACCAAAAATCATTGGTAAAACATTT   699
```

```
XI3Ex5-Ex9      GTATTTGCTCTATTTGGTGCATTCTACTTCTGGACACCAAAAATCATTGGTAAAACATTT    697
SRZ2Ex5-9       GTATTTGCTCTATTTGGTGCATTCTACTTCTGGACACCAAAAATCATTGGTAAAACATTT    719
XII2Ex5-Ex9     GTATTTGCTCTATTTGGTGCATTCTACTTCTGGACACCAAAAATCATTGGTAAAACATTA    688
                ***********************************************************:

XI2Ex5-Ex9      AACGAGAACCTAGGACGAATCCATTTCTGGACACTATTTGTAGGAGTTAACCTAACATTT    759
XI3Ex5-Ex9      AACGAGAACCTAGGACGAATCCATTTCTGGACACTATTTGTAGGAGTTAACCTAACATTT    757
SRZ2Ex5-9       AACGAGAACCTAGGACGAATCCATTTCTGGACACTATTTGTAGGAGTTAACCTAACATTT    779
XII2Ex5-Ex9     AACGAGAACCTAGGACGAATCCAT-TCTGGACCCTATTTGTAGGAGTAA-CCTAACATTT    746
                ************************ *******.**************:* **********

XI2Ex5-Ex9      TCCGCAAACATTTTAAA-------------------------------------------    776
XI3Ex5-Ex9      TCCCCAAAC-NTTT----------------------------------------------    770
SRZ2Ex5-9       TTCCCGCAACATTTTCTAGGTCTTGGTGGTATGCCACGGCGGATTCCAGACTATCCAGAT    839
XII2Ex5-Ex9     TTCGCC-ACCATTTTATTA-----------------------------------------    764
                * * *  *.  ***
```

CLUSTAL O(1.2.4) multiple sequence alignment

```
SRZ2Ex5-9       ---------------------------------AITMLLTDRNFNTSFYDPAGGGDPILY 27
XI2Ex5-Ex9      RNPQMTLHKLPLFVWAIFVTAILLLLSLPVLAGAITMLLTDRNFNTSFYDPAGGGDPILY 60
XI3Ex5-Ex9      -TPRMTLHKLPLFVWAIFVTAILLLLSLPVLAGAITMLLTDRNFNTSFYDPAGGGDPILY 59
XII2            ----MTLHKLPLFVWAIFVTAILLLLSLPVLAGAITMLLTDRNFNTSFYDPAGGGDPILY 56
                                                 ***************************

SRZ2Ex5-9       QHLFSFFGHPEVYILIIPGFGMVSHIVSAFSGKPVFGYLGMVYAMFSIGILGFLVWSHHM 87
XI2Ex5-Ex9      QHLFWFFGHPEVYILIIPGFGMVSHIVSAFSGKPVFGYLGMVYAMFSIGILGFLVWSHHM 120
XI3Ex5-Ex9      QHLFWFFGHPEVYILIIPGFGMVSHIVSAFSGKPVFGYLGMVYAMFSIGILGFLVWSHHM 119
XII2            QHLFWFFGHPEVYILIIPGFGMVSHIVSAFSGKPVFGYLGMVYAMFSIGILGFLVWSHHM 116
                **** *******************************************************

SRZ2Ex5-9       YAVGLDVDTRAYFTAATMIIAVPTGIKIFSWLATLYGGSLRITTPMLFALGFIALFTIGG 147
XI2Ex5-Ex9      YAVGLDVDTRAYFTAATMIIAVPTGIKIFSWLATLYGGSLRITTPMLFALGFIALFTIGG 180
XI3Ex5-Ex9      YAVGLDVDTRAYFTAATMIIAVPTGIKIFSWLATLYGGSLRITTPMLFALGFIALFTIGG 179
XII2            YAVGLDVDTRAYFTAATMIIAVPTGIKIFSWLATLYGGSLRITTPMLFALGFIALFTIGG 176
                ************************************************************

SRZ2Ex5-9       LTGVILANASLDVALHDTYYVVAHFHYVLSMGAVFALFGAFYFWTPKIIGKTFNENLGRI 207
XI2Ex5-Ex9      LTGVILANASLDVALHDTYYVVAHFHYVLSMGAVFALFGAFYFWTPKIIGKTFNENLGRI 240
XI3Ex5-Ex9      LTGVILANASLDVALHDTYYVVAHFHYVLSMGAVFALFGAFYFWTPKIIGKTFNENLGRI 239
XII2            LTGVILANASLDVALHDTYYVVAHFHYVLSMGAVFALFGAFYFWTPKIIGKTLNENLGRI 236
                ****************************************************:*******

SRZ2Ex5-9       HFWTLFVGVNLTFFPQHFLGLGGMPRRIPDYPDAFAAWNAISSFGSLVSVVATALFGYII 267
XI2Ex5-Ex9      HFWTLFVGVNLTFSANIL------------------------------------------ 258
XI3Ex5-Ex9      HFWTLFVGVNLTFSPNX------------------------------------------- 256
XII2            HSGPYL*E*PNIFRHHFI------------------------------------------ 252
                *    :      *  :
```
