# Supplementary material for: Comparative mitogenomic analysis of Sporisorium reilianum f. sp. zeae suggests recombination events during its evolutionary history
Source: Front Physiol. 2024 Sep 6;15:1264359. doi: 10.3389/fphys.2024.1264359 (PMC11413489; doi:10.3389/fphys.2024.1264359)
Supplement: Supplementary file 8 [file DataSheet1.PDF]

SUPPLEMENTARY TABLE 1 – Primers used in this study.

Primer Identifier (oHM#)	Primer Sequence (5’ → 3’)	Purpose
112	GCCGACGGTCTTTATACGAA	Diagnostic
113	GCGCCTTCTTTTCTTGAATG	Diagnostic
114	TCTTGCTGAAGGACCCCTTA	Diagnostic
115	TAACGGAAAAATGCGAGGTC	Diagnostic
116	TGGGGACTCTATCTTCATCCA	Diagnostic
119	TAGCTCGTTTTCGGCCTTTA	Diagnostic
120	CGAAACCCAGGAATGACACT	Diagnostic
121	AAAGCGCGATCTACAAGACC	Diagnostic
122	TTTGCCCAACATACCCTGAT	Diagnostic
127	TCCCCTTTTTATTAGCTGAGCA	Diagnostic
128	TCAAGATTTTTGGCAATTACAATG	Diagnostic
131	CACCACAAATCCAATGACTGA	Diagnostic
133	TGGGGACTCTATCTTCATCCA	Primer walking *cox1* polymorphism
134	GGCAATTCGAAATGGGATAG	Primer walking *cox1* polymorphism
135	CACCCTCTTTATGAAAAATGACAA	Primer walking *cox1* polymorphism
136	AGGCTACACGCGAAACAACT	Primer walking *cox1* polymorphism

137	AAGCTACGATCCGGAGCTAAA	Primer walking *cox1* polymorphism
138	TCAATGGTCTGTGCAAAGTAAGA	Primer walking *cox1* polymorphism
139	CGTAGGGCATACAGATCCTCA	Primer walking *cox1* polymorphism
157	TCTGGGTAATCAGGGATTCG	Diagnostic
158	ACGCGGTAGTTCTGTTGACC	Diagnostic
159	CAATAGCGGTTTGTCCATGA	Diagnostic
160	TCATTGTATGTGCTAACGCTAGAT	Diagnostic
161	AAAATGTTGCGGGAAAAATG	Diagnostic
162	TGCTATCAGATGCTTGGATGA	Diagnostic
163	TTCCGGTCTGTTAGTAGCATTG	Diagnostic
164	GCATGTCCTTCTCCCCCTAT	Diagnostic
SRZ2mtEx2Ex3F	AATTCACCCGATGCAACTCT	Diagnostic
SRZ2mtEx2Ex3R	TACACATGCTCCAAGCGGTA	Diagnostic
SRZ2mtNearEx3F	CGCTCGTGTCCGGTTTAT	Diagnostic
SRZ2mtNearEx3R	TATTGTGAAATATAGAGTTGCATCG	Diagnostic
SRZ2mtEx2Ex5F	TGGTTTCCTGCTGATTATCCA	Diagnostic
SRZ2mtEx2Ex5R	TCTCGTTCCGGTGCTTATCT	Diagnostic
